# Outer membrane permeability of *Pseudomonas aeruginosa* through β-lactams: new evidence on the role of OprD and OpdP porins in antibiotic resistance

**DOI:** 10.1128/spectrum.00495-24

**Published:** 2025-03-04

**Authors:** Francesco Amisano, Paola Mercuri, Steven Fanara, Olivier Verlaine, Patrick Motte, Jean Marie Frère, Marc Hanikenne, Moreno Galleni

**Affiliations:** 1 InBioS, Center for Protein Engineering, Biological Macromolecules, Department of Life Sciences, University of Liège, Liège, Belgium; 2 InBioS - PhytoSystems, Functional Genomics and Plant Molecular Imaging and Centre for Assistance in Technology of Microscopy (CAREm), University of Liège, Liège, Belgium; 3 InBioS-PhytoSystems, Translational Plant Biology, University of Liège, Liège, Belgium; University of L'Aquila, L'Aquila, Italy

**Keywords:** OpdP porin, external membrane permeability, *Pseudomonas aeruginosa*, porins

## Abstract

Gram-negative bacteria are a major concern for public health, particularly due to the continuous rise of antibiotic resistance. A major factor that helps the development of resistance is the outer membrane that is essential since it acts as a strong permeability barrier to many antibiotics that are effective against other bacteria. In this study, we determine the specific permeability coefficients for various antibiotics in *Pseudomonas aeruginosa* strains, which differ from each other for their porin expressions. We showed that OprD and OpdP porins contribute both to internalize meropenem and biapenem. Using qRT-PCR, we demonstrated that their expression is dependent of the various phases of cellular growth. We were able to show how the OpdP porin is less expressed in exponential growth phases, while it tends to be produced when the bacterial culture enters into the latent phase, in an inversely proportional way compared to the OprD porin. The deletion of the OpdP porin, in the presence of meropenem at concentrations equivalent to the MIC values, contributes to the selection of carbapenem-resistant strains. Therefore, the presence of mutations/deletions of the OpdP porin should receive greater consideration from a clinical point of view as the use of meropenem at nonoptimal concentrations could lead to the appearance of resistance phenotypes.

**IMPORTANCE:**

Carbapenem-resistant strains of *Pseudomonas aeruginosa* are among the major threats to public health. The permeability of the outer membrane for the β-lactam antibiotics is one of the major factors that reduce the activity of the antibiotics. In this study, we measure the low permeability coefficient of the *P. aeruginosa* outer membrane to β-lactams. The methodology we develop to determine the permeability can be applied to other antibiotic families and/or pathogens.

## INTRODUCTION

The gram-negative rod-shaped γ-proteobacterium *Pseudomonas aeruginosa* (*P. aeruginosa*) is a ubiquitous and opportunistic pathogen responsible for life-threatening infections, especially in immune-compromised patients, such as those with ventilator-associated pneumonia (VAP) or with urinary tract infections (UTIs) and is the leading cause of respiratory tract infections (RTIs) in cystic fibrosis patients (1–4). It is intrinsically resistant to different classes of antibiotics, and the few available therapeutical options include some β-lactam compounds, often delivered in combination with β-lactamase inhibitors, such as piperacillin/tazobactam, ceftazidime/avibactam, and imipenem/relebactam (5–8). Unfortunately, this bacterium has developed different mechanisms of resistance. In the case of β-lactam antibiotics, the major resistance mechanism is the production of enzymes (β-lactamases) that can hydrolyze the β-lactam ring (3, 9). *P. aeruginosa* carries a chromosomally encoded and inducible AmpC β-lactamase (10). In addition, it can acquire extended-spectrum β-lactamases (ESBLs) and/or metallo-β-lactamases (MBLs), which confer resistance to carbapenems (11–13).

Moreover, *P. aeruginosa* is characterized by a low outer membrane permeability to β-lactams, due to the presence of OprF, a nonspecific pore, homologous to OmpC and OmpF from *Escherichia coli*. This porin is, indeed, present into two conformers, and the closed fraction represents approximatively 95% of the total OprF expressed by the bacteria, leaving a minority in the open state, and its involvement in the permeation is still debated (14, 15).

As a consequence, hydrophilic nutrients and antibiotic internalization are mediated by a wide number of substrate selective channels (15).

Interplay with other resistance mechanisms, like the efflux pump upregulation, can extrude a wide variety of antibiotics present in the cytoplasmic or periplasmic space of the bacteria. In particular, increased expression of MexAB-OprM contributes to β-lactam resistance, as commonly found in clinical multiresistant isolates (16–18).

It is well documented that a decrease in the outer membrane permeability can be mediated by an altered expression of OprD. This porin facilitates basic amino acid uptake and influences antibiotic sensitivity, primarily to imipenem, due to the structural homology between arginine and the C2 antibiotic lateral chain (19, 20).

*P. aeruginosa* possesses 18 OprD homologs, characterized by their structural similarities and substrate specificities. They belong to the family of outer membrane carboxylate channel (Occ).

Within this family, two subgroups are identified: the OprD (or OccD) and the OpdK (or OccK) subfamilies (15, 21, 22).

OpdP (OccD3) shows the highest sequence identity with OprD (51%). It is associated with glycine–glutamate dipeptide translocation, and it has been assumed to be involved in meropenem uptake, although a clear phenotypic resistance profile in deletion mutants has not been determined (21, 23–26). This porin belongs to the *dppA4BCDF* operon, encoding the ABC machinery responsible for the utilization of dipeptides during the stationary phase, increasing the bacterial metabolic versatility. Its expression is controlled by the PsdR regulator (27, 28). Recent studies have shown that *psdR* is prone to acquire mutations, but their impact on the OpdP expression has not yet been described (29–31).

The research for antibiotic-specific channels has been directed to other porins belonging to the Occ family. For instance, in the study by Isabella and coworkers, porins selected for their expression quantified by RNAseq analysis after growth in minimal medium were hypothesized to be involved in the entry of antibiotics. The study identified the OpdC (OccD2), OpdT (OccD4), and OpdB (OccD7) porins as possible candidates. However, a *P. aeruginosa* isogenic mutant where the genes encoding three porins were deleted, together with *opdP* and *oprD,* did not display a modification of the antibiotic resistance profile compared to the single *oprD* mutant (24). Even a *P. aeruginosa* strain stripped of 40 porins resulted in MICs comparable to those of the single *oprD* knockout, suggesting the presence of alternative translocation pathways, independent of porins (32). For this reason, the MIC determination of *P. aeruginosa* isolates does not reflect the real pattern of porin expression and does not exhibit a reliable predictive value for bacterial permeability.

Therefore, an improved understanding of β-lactam translocation mechanisms in gram-negative bacteria might help in the design of new molecules, formulated also on the basis of their abilities to cross the outer membrane barrier.

Different methods have been proposed to study the outer membrane permeability in *P. aeruginosa*, starting from the pioneering work by Zimmermann and Rosselet (33). Comparing the periplasmic β-lactam hydrolysis of intact cells with the one obtained by a lysate made it possible to determine the outer membrane permeability coefficients for *P. aeruginosa* (34). Unfortunately, this method turned out to be poorly reproducible due to the contribution of efflux pumps that interfere with antibiotic accumulation (35).

The use of radio-labeled β-lactams has been proposed as an alternative method for the quantification of the antibiotic periplasmic concentration (36). However, this method is limited by the difficulty of obtaining a wide set of radioactive compounds.

Whole-cell analytical techniques, including mass spectrometry-based analysis, allow the quantification of the variation in extracellular antibiotics or the measurement of the direct accumulation in the periplasm (37–39), but they are highly time-consuming.

Another approach being pursued was the study of single porin permeation properties using different techniques such as the liposome swelling assay, electrophysiology, or molecular dynamics simulations. They have importantly contributed to the definition of the specific role of single porins, the determination of their specific conductance, and have identified the consequences that mutations may have on translocation properties (40–44). Nevertheless, the study of the single porin properties undoubtedly failed to comprehend the complexity of the bacterial response to antibiotics, given that synergic effects are not noticeable.

In a first approach, we determined the *P. aeruginosa* outer membrane permeability toward β-lactam, as described below. This method exploits the property of BlaR-CTD, a soluble penicillin-binding protein that displays a high affinity for β-lactams. Its expression in the periplasm allows an accurate estimation of the quantity of the antibiotic that permeates through the outer membrane (45).

Unlike the previously mentioned methods, the study of single or multiple porin(s) isogenic mutants can elucidate the role of a single porin or the presence of any synergic effect in double or multiple knockout strains for a broad variety of β-lactams, and this was the first aim of our research.

The second goal of this study was to ascertain the real contribution of OpdP in carbapenem resistance, especially under stress conditions. To the best of our knowledge, we were the first to investigate the expression of different porins during different bacterial growth phases by means of qRT-PCR, and we believe that this is a useful tool to broaden our understanding of the response to antibiotic therapy.

Our third goal was to verify whether the single deletion of the OpdP porin confers a selective advantage in developing a phenotype of carbapenem resistance. To this end, we performed a multistep resistance experiment using meropenem at sub-minimum inhibitory concentrations and subsequently analyzed the resistant mutants thus obtained.

Finally, we performed whole-genome sequencing on selected strains to clarify the specific resistance genotype.

## RESULTS AND DISCUSSION

### Antibiotic resistance determination and mutant selection

Different subclasses of β-lactams were tested to evaluate the MIC variations associated to porin deletions, and the resistance profiles of *P. aeruginosa* strains are reported in Table 1.

**TABLE 1:** MIC values for the different *P. aeruginosa* strains^*a*^^,^^*b*^

Antibiotics (µg/mL)	*P. aeruginosa* MICs (µg/mL)																	
	CLSI Standard	CLSI Susc.	ATCC 27853	PAO1	PAO1-Jap	TNP004	ARC545	ARC5990 (*Δoprd*)	ARC5170 (*Δopdp*)	ARC5782 (*Δoprd, ΔopdP*)	ARC5998 (Δ5porins)	LG01	LG02	LG03	LG04	LG05	LG06	LG07
Ampicillin	NA	NA	2,000	2,000	2,000	1,000	2,000	1,000	1,000	1,000	1,000	2,000	2,000	2,000	ND	ND	ND	ND
Benzylpenicillin	NA	NA	>2,000	>2,000	>2,000	>2,000	>2,000	>2,000	>2,000	>2,000	>2,000	>2,000	>2,000	>2,000	ND	ND	ND	ND
Piperacillin	1–8	≤16	2	2	2	2	2	2	2	2	2	8	4	8	ND	ND	ND	ND
Cefalotin	NA	NA	>2,000	>2,000	>2,000	>2,000	>2,000	>2,000	>2,000	>2,000	>2,000	ND	ND	ND	ND	ND	ND	ND
Cephaloridine	NA	NA	>2,000	>2,000	>2,000	>2,000	>2,000	>2,000	>2,000	>2,000	>2,000	ND	ND	ND	ND	ND	ND	ND
Cefoxitin	NA	NA	1,000	1,000	1,000	500	1,000	1,000	1,000	1,000	1,000	ND	ND	ND	ND	ND	ND	ND
Cefuroxime	NA	NA	250	250	250	250	250	500	500	500	500	>500	>500	>500	ND	ND	ND	ND
Cefotaxime	8–32	NA	16	16	16	8	16	16	16	16	16	32	16	32	ND	ND	ND	ND
Ceftazidime	1–4	≤8	1	1	1	2	1	1	1	1	1	2	1	2	ND	ND	ND	ND
Cefepime	0.5–4	≤8	1	1	1	0.5	1	1	1	1	1	2	2	2	ND	ND	ND	ND
Imipenem	1–4	≤2	1	1	1	**8**	1	**8**	1	**8**	**8**	**16**	**16**	**8**	**16**	**16**	**16**	**16**
Meropenem	0.12–1	≤2	0.5	0.5	0.5	**2**	0.5	**4**	0.5	**4**	**4**	**8**	**4**	**8**	**4**	**4**	**4**	**4**
Ertapenem	2–8	NA	8	8	8	**32**	8	**32**	8	**32**	**32**	**64**	**64**	**64**	**64**	**64**	**64**	**64**
Biapenem	0.5–2	NA	0.5	0.5	0.5	**4**	0.5	**4**	0.5	**4**	**4**	**4**	**4**	**4**	**4**	**4**	**4**	**4**
Doripenem	0.12–0.5	≤2	0.25	0.25	0.25	**1**	0.25	**1**	0.25	**1**	**1**	**2**	**2**	**2**	**2**	**2**	**2**	**2**
Tetracycline	8–32	≤4	8	8	8	8	8	8	8	8	8	16	8	16	ND	ND	ND	ND
Gentamicin	0.5–2	≤4	1	1	1	2	1	2	2	2	2	ND	ND	ND	ND	ND	ND	ND

^*a*^ CLSI standard refers to acceptable limits for quality control strains used to monitor the accuracy of MICs. CLSI susc. refers to the MIC susceptibility breakpoints interpreted by CLSI (46). NA: not available, ND not determined.

^*b*^ The bold characters represent the names of the *Pseudomonas* strains utilized in this study.

MIC values for carbapenems were increased in all the strains where OprD was deleted and also in TNP004, described to downregulate OprD expression (47). The deletions of the other porins did not change the resistance phenotype and did not suggest any synergic effect for the antibiotic tested.

We further obtained *P. aeruginosa* mutant strains, derived from *P. aeruginosa* PAO1 and *P. aeruginosa* ARC5170 (PAO1*ΔopdP*), with the help of a multistep resistance experiment.

Colonies were cultured and then plated at different growth phases at sub-MIC meropenem concentrations. We registered the appearance of resistant colonies for both strains and at the different growth phases tested, but ARC5170 proved to be the most adept at acquiring the ability to grow in the presence of meropenem, 30 times more frequently than for the other strain; in particular, we found approximatily 600 colonies derived from ARC5170, while only 20 were derived from PAO1.

We selected *P. aeruginosa* LG01, derived from PAO1, and six mutants derived from ARC5170 named *P. aeruginosa* LG02-LG07; determined their MICs; and observed that all the strains presented a stable resistance profile characterized by a reduced sensitivity to carbapenems, identical to that of the strains deprived of OprD (Table 1).

### OprD sequencing and whole-genome sequencing

*P. aeruginosa* TNP004 was described to produce an undetectable amount of OprD (47), but the cause underlying this downregulation has not been elucidated yet. For this reason, we sequenced the *oprD* gene and observed that it contained a single-nucleotide mutation (T1301C) yielding the Leu434Pro mutation in OprD, a modification in a transmembrane domain that increases the instability of the porin (GenBank accession number OR069747).

We were also interested in assessing whether the carbapenem resistance found in the strains selected under meropenem pressure was attributable to *oprD* mutations and consequently performed Sanger sequencing. For *P. aeruginosa* LG01, the insertion of one cytosine between positions 1,205 and 1,206 was found to induce a frameshift in the *oprD* open reading frame (GenBank accession number OR069748). LG02 is characterized by a single-nucleotide substitution (G1017A), introducing a premature STOP codon (GenBank accession number OR069749). The strains LG04, LG05, LG06, and LG07 shared the same nucleotide deletion in position 1,291 (G), resulting in a frameshift and in the synthesis of a truncated porin (GenBank accession number OR069750). Figure S3 summarizes the alignments of the different *oprD* mutated genes. Interestingly, *P. aeruginosa* LG03 did not exhibit any mutation when compared to the *oprD* wild-type gene, despite a carbapenem resistance profile similar to that of the other *oprD* mutants.

The whole-genome sequences of *P. aeruginosa* TNP004 and LG03 were determined to identify other mutations that could lead to the carbapenem resistance genotype associated to those strains. Due to the report of many polymorphisms in different PAO1 reference strains diffused worldwide (48), PAO1-Jap and ARC545, parental strains of TNP004 and ARC5170, respectively, were sequenced in order to exclude the role of mutations already present in these strains for carbapenem resistance observed in TNP004 and LG03.

**Fig 1:**
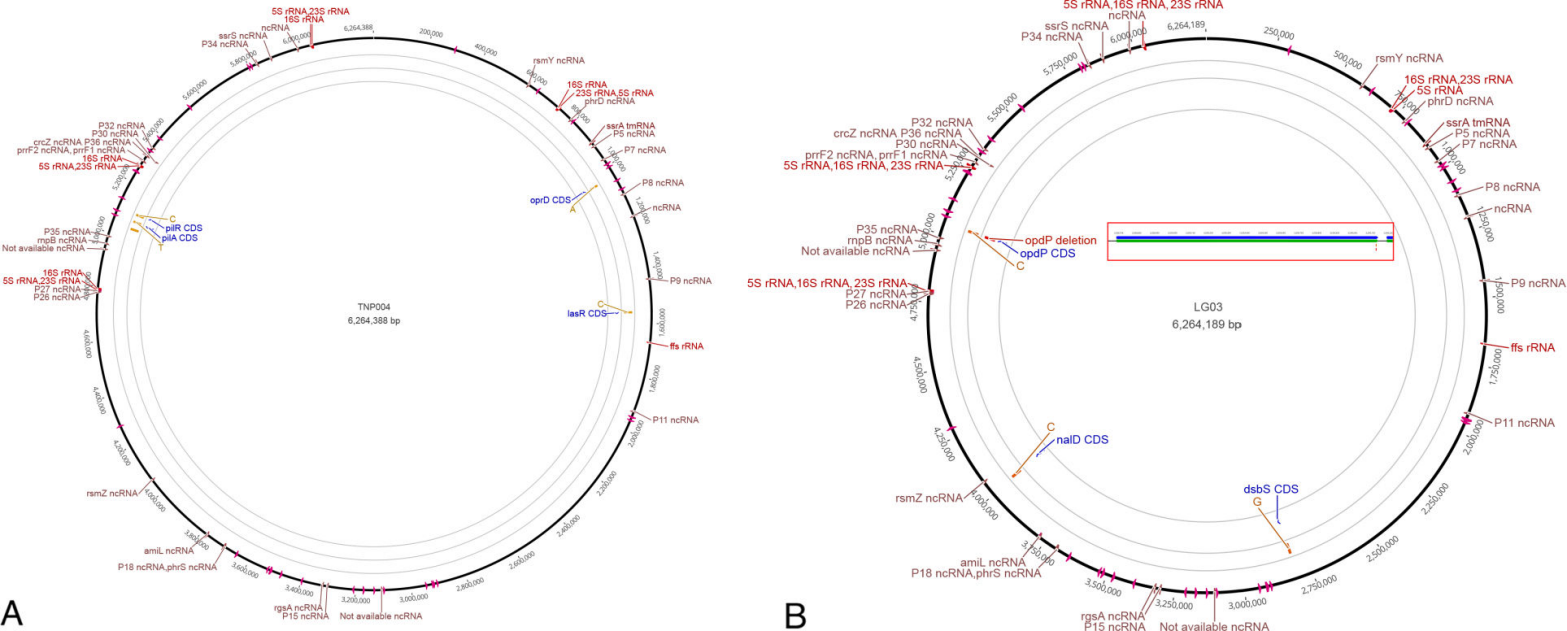
Circular visualization of the genome assembly obtained using the software Geneious (version R10). (A) The genome sequence of TNP004 was compared to that of its parental strain PAO1-Jap. The mutations between the genomes are shown. (B) Genome sequence of LG03 was compared with that of its parental strain ARC545. The opdP deletion is highlighted, and the other observed mutations are shown.

The nucleotide sequences were deposited in the GenBank database, Bioproject PRJNA985251 under accession number SAMN35794375-8, while the observed mutations are reported in Fig. 1.

TNP004, besides the nucleotide substitution in *oprD*, presented other mutations including a deletion in *fliF* and two point mutations in *lasR* and *pilR*. However, these genes have not been described to be involved in the regulation of OprD expression, and so we concluded that the mere amino acid substitution in OprD causes the lack of porin’s expression.

We also observed that, when compared to the reference PAO1, LG03 exhibited two single-nucleotide polymorphisms in *nalD* and *dsbS* in addition to the *opdP* deletion.

*dsbS* was recently described as a histidine-kinase sensor that acts together with the cognate response regulator *dsbR* in copper homeostasis (49). The copper-induced response has been previously shown to reduce the OprD expression through a regulation mediated by different two-component systems, such as *czcRS* and *copRS* (50, 51). It is reasonable to speculate that also this third copper regulation system might govern OprD expression. *nalD* is a transcriptional repressor of cellular efflux whose mutations have been correlated with MexAB-OprM overexpression (52); the upregulation of another efflux pump system, MexEF-OprN, mediated by the regulator *mexT* is known to decrease OprD expression (53). However, although many strains have been described to exhibit mutations in *nalD* causing MexAB-OprM overexpression, there is no evidence in the literature that these mutations could directly influence OprD.

The sequencing has therefore revealed that a single mutation can cause carbapenem resistance in TNP004, while *oprD* mutations for six of the seven mutants selected under meropenem pressure alter the correct OprD synthesis, giving rise to increased resistance to carbapenems. Furthermore, the whole-genome sequencing has revealed the presence in LG03 of two mutations that contribute to carbapenem resistance, but their actual contribution requires further investigations.

### Western blot

**Fig 2:**
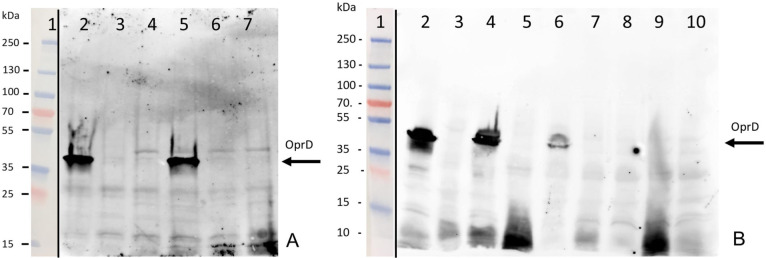
OprD detection performed by Western Blot in the following strains: (A). 1, protein marker; **2**,PAO1; 3,TNP004; **4**, ARC5990 (PAO1.ΔoprD); **5**, ARC5170 (PAO1ΔopdP); **6**, ARC5782 (PAO1ΔoprD, ΔopdP) and **7**, ARC5998 (PAO1ΔoprD, ΔopdP,ΔораB, ΔopdC, and ΔopdT). (B). **1**, protein marker; **2**, PAO1; 3, LG01; **4**, ARC5170 (PAO1ΔopdP); **5**, LG02; **6**, LG03; **7**, LG04; **8**, LG05; **9**, LG06 and **10**, LG07. The porin OprD is marked by an arrow. Protein markers’ pictures were put aside the chemiluminescence scans.

The expression of OprD in different mutant strains was verified by means of Western Blot (Fig. 2). As assumed, we confirmed the absence of the porin not only in the deleted mutants but also in the *oprD* mutant TNP004 and in the LG01, LG02, and LG04-LG07 strains, confirming the sequencing results. Interestingly, we could observe that LG03 was characterized by a downregulation of OprD, thus resulting in a consistent resistance profile to carbapenems.

### Growth curves

**Fig 3:**
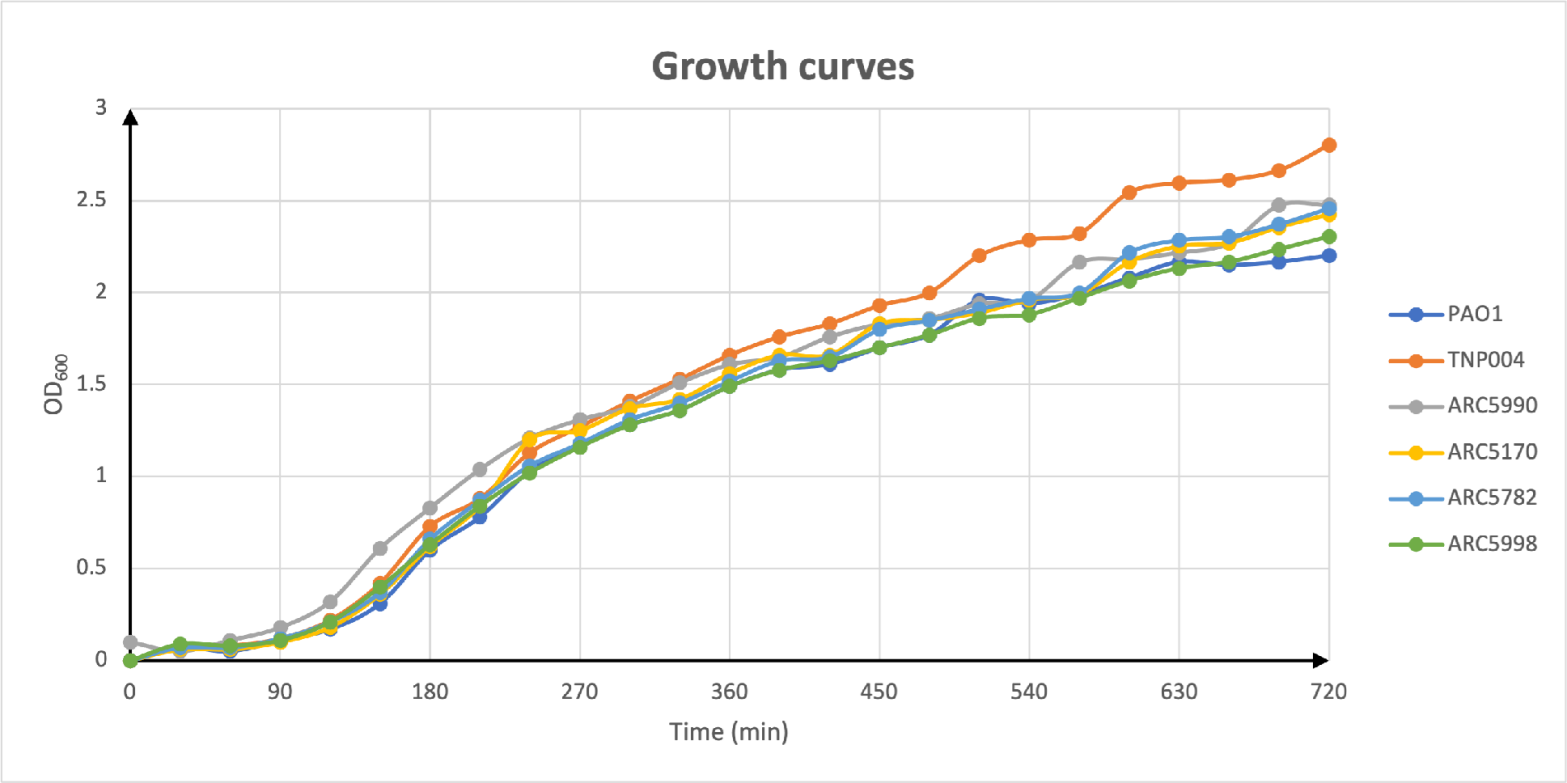
Growth curves of *P. aeruginosa* strains in LB cultures.

The planktonic growth in the LB medium of different porin mutants was compared to that of the wild-type PAO1 (Fig. 3). Remarkably, we did not observe an important delay in the growth of the different tested mutants compared to *P. aeruginosa* PAO1. Even ARC5998, with five different porins deleted, was able to grow in the LB medium at a rate similar to that of the PAO1 wild-type strain.

A possibly relevant difference is shown only in TNP004 where the cell density is slightly larger compared to the other strains. We could so assess that the lack of one or more porins does not influence bacterial growth, at least in the LB medium.

### Permeability coefficient determination

A better understanding of the permeability of gram-negative bacteria, and in particular of *P. aeruginosa*, is an important factor for better directing the search for new antibiotics (54, 55).

While the role of the OprD porin in imipenem resistance is now widely understood (20, 56), the permeation of other antibiotics through the outer membrane remains difficult to interpret (15, 26, 57); indeed, antibiotic diffusion due to other porins does not seem to be sufficient to explain their uptake (32).

With this aim in mind, we adapted a previously described protocol (45) to quantify the β-lactam translocation into *P. aeruginosa* periplasm in order to determine the permeability coefficients of the outer membrane to different β-lactams. We performed validations to exclude major interfering events that could occur during measurements that include the following: i) the absence of MIC variation as a consequence of BlaR-CTD periplasmic production, ii) the verification of BlaR-CTD high affinity for the β-lactams tested, and iii) the absence of significant AmpC induction in the presence of β-lactams.

We transformed *P. aeruginosa* PAO1, ARC5990 (PAO1*ΔoprD*), ARC5170 (PAO1*ΔopdP*), ARC5782 (PAO1*ΔoprD*, *ΔopdP*), ARC5998 (PAO1 five porins mutant) with plasmid pKT240blaR, and TNP004 (PAO1 **↓**OprD) with pKT240blaR-gen, a derived plasmid carrying gentamicin resistance, to produce BlaR-CTD in the periplasm of these strains.

We compared the MIC profiles of the strains producing BlaR-CTD or not. We also transformed PAO1 with an empty vector (pKT240neg) as a control to compare the effects induced by the plasmid alone to those caused by BlaR-CTD expression.

As reported in Table 2, the presence of BlaR in the periplasmic space did not alter the MIC values of the host bacteria with the exception of three compounds, piperacillin, ceftazidime, and cefepime, that presented unexpected MIC increases ranging from twofold to fourfold dilutions when compared to the *blaR*-devoid strain.

**TABLE 2:** MIC values of the different *P. aeruginosa* strains producing or not BlaR-CTD^*a*^^,^^*b*^

Antibiotics (µg/mL)	*P. aeruginosa* MICs (µg/mL)														
	CLSI Standard	CLSI Susc.	PAO1	PAO1 pKT240neg	PAO1 pKT240blaR	TNP004	TNP004 pKT240blaR	ARC5990 (*Δoprd*)	ARC5990 pKT240blaR	ARC5170 (*Δopdp*)	ARC5170 pKT240blaR	ARC5782 (*Δoprd, ΔopdP*)	ARC5782 pKT240blaR	ARC5998 (Δ5porins)	ARC5998 pKT240blaR
Ampicillin	NA	NA	2,000	1,000	1,000	1,000	500	1,000	1,000	1,000	1,000	1,000	1,000	1,000	1,000
Benzylpenicillin	NA	NA	>2,000	>2,000	2,000	>2,000	>2,000	>2,000	>2,000	>2,000	>2,000	>2,000	>2,000	>2,000	>2,000
Piperacillin	1–8	≤16	2	1	4	2	4	2	**8**	2	**8**	2	**8**	2	**8**
Cefalotin	NA	NA	>2,000	>2,000	>2,000	>2,000	2,000	>2,000	>2,000	>2,000	>2,000	>2,000	>2,000	>2,000	>2,000
Cephaloridine	NA	NA	>2,000	>2,000	>2,000	>2,000	2,000	>2,000	>2,000	>2,000	>2,000	>2,000	>2,000	>2,000	>2,000
Cefoxitin	NA	NA	1,000	1,000	500	500	500	1,000	1,000	1,000	1,000	1,000	1,000	1,000	1,000
Cefuroxime	NA	NA	250	250	250	250	250	500	500	500	500	500	500	500	500
Cefotaxime	8–32	NA	16	16	16	8	16	16	16	16	16	16	16	16	16
Ceftazidime	1–4	≤8	1	1	**16**	2	**32**	1	**16**	1	**16**	1	**16**	1	**16**
Cefepime	0.5–4	≤8	1	1	**4**	1	**4**	1	**4**	1	**4**	1	**4**	1	**4**
Imipenem	1–4	≤2	1	1	1	8	8	8	8	1	1	8	8	8	8
Meropenem	0.12–1	≤2	0.5	0.5	0.5	2	2	4	4	0.5	0.5	4	4	4	4
Ertapenem	2–8	NA	8	8	8	32	32	32	32	8	8	32	32	32	32
Biapenem	0.5–2	NA	0.5	0.5	0.5	4	4	4	4	0.5	0.5	4	4	4	4
Doripenem	0.12–0.5	≤2	0.25	0.25	0.25	1	1	1	1	0.25	0.25	1	1	1	1
Tetracycline	8–32	≤4	8	>128	>128	8	>128	8	>128	8	>128	8	>128	8	>128
Gentamicin	0.5–2	≤4	1	1	2	2	16	2	2	2	2	2	2	2	2

^*a*^ The values previously obtained for the non-transformed strains are reported. The CLSI standard refers to acceptable limits for quality control strains used to monitor the accuracy of MICs. CLSI susc. refers to the MIC susceptibility breakpoints interpreted by CLSI (46). NA: not available, ND not determined.

^*b*^ The bold characters represent the names of the Pseudomonas strains utilized in this study.

This phenomenon could be explained by a lower affinity of these three antibiotics for natural PBPs compared to the other antibiotics tested. A similar conclusion was made by Montaner and coworkers studying the effect of AmpC hyperexpression (58). The periplasmic BlaR-CTD production, similarly to AmpC hydrolysis, might reduce the intracellular concentration of these three antibiotics to a limit level that would change the PBP occupancy causing increases in MICs, or, equivalently, might induce AmpC expression. However, whatever the exact cause, we excluded these antibiotics from our permeability study and preferred to concentrate on antibiotics whose MICs were not altered by BlaR-CTD expression.

We successively measured the acylation constants of BlaR-CTD with the different β-lactam antibiotics (Table 3). Our data confirmed that the formation of a stable acyl-enzyme was not a rate-limiting step in our experiments (k_2_/K′ >0.02 µM^−1^ s^−1^).

**TABLE 3:** Acylation rate constant (*K_2_/K*) for the different tested antibiotics

Antibiotic	*k_2_/K* (μM^−1^ · s^−1^)	Reference
Nitrocefin	3.6 ± 0.3	This study
Benzylpenicillin	8.7 ± 1.1	(57)
Ampicillin	1.3 ± 0.1	(57)
Cephaloridine	5.9 ± 0.2	(57)
Cefoxitin	0.06 ± 0.02	(57)
Cefuroxime	0.02 ± 0.005	(57)
Cefotaxime	0.04 ± 0.003	(57)
Imipenem	0.8 ± 0.2	This study
Meropenem	0.8 ± 0.2	This study
Ertapenem	1.1 ± 0.2	This study
Biapenem	1.4 ± 0.3	This study
Doripenem	1.7 ± 0.3	This study

We finally verified that the expression of the chromosome-encoded AmpC β-lactamase did not affect the detection of labeled BlaR-CTD. To do so, we determined the level of production of the AmpC β-lactamase in the different *P. aeruginosa* PAO1 cultures. The concentration of the antibiotic added to the culture was identical to its maximum concentration used during the permeability assay, and the incubation times for different β-lactams were equal to the maximum duration of the permeability measurements for the specific antibiotic. We, therefore, determined the AmpC concentration in the function of the incubation time (Table 4).

**TABLE 4:** Periplasmic AmpC concentration and specific activity of the AmpC β-lactamase in the presence or absence (/) of β-lactams for the different *P. aeruginosa* cultures^*a*^

Incubation time (min)	Antibiotic (final concentration μM)	Periplasmic AmpC (mg/L)	Specific activity (μmol · min^−1^ · mg^−1^)
12	Imipenem (0.02)	0.029	0.07
	Doripenem (1)	0.024	0.06
	Biapenem (0.04)	0.020	0.05
	/	0.026	0.06
20	Meropenem (2)	0.020	0.05
	/	0.042	0.10
30	Ampicillin (20)	0.026	0.05
	Benzylpenicillin (40)	0.027	0.08
	Cephaloridine (7.5)	0.024	0.08
	Ertapenem (7.5)	0.073	0.25
	/	0.029	0.06
40	Cefoxitin (30)	0.49	1.25
	Cefuroxime (60)	0.018	0.03
	Cefotaxime (60)	0.021	0.03
	/	0.017	0.03
360	Ampicillin (50)	22.6	1.43
	Cefoxitin (30)	90.4	5.76
	/	4.88	0.31

^*a*^ The activity was followed by nitrocefin hydrolysis. (/) refers to a negative culture where no antibiotic was added.

We showed that the specific activity of the chromosome-encoded AmpC remained similar to those obtained for the negative controls. Nevertheless, ertapenem showed a minor increase in the AmpC activity (fourfold compared to the negative control). The validity of the assay to detect increased expression of the β-lactamase was demonstrated by verifying the strong AmpC induction after a longer incubation (6 hours) in the presence of ampicillin and cefoxitin, known to be good AmpC inductors (59).

Antibiotic penetration was assessed in planktonic cultures grown in LB, and the assay was performed during the late exponential growth phase (A_600_ approximatively 1.6), thus excluding an increase in periplasmic BlaR-CTD due to bacterial duplication during the short duration of the analysis (maximum 40 minutes).

We first determined the permeability coefficients for different β-lactams in *P. aeruginosa* PAO1, and the results are reported in Table 5 (60–62). One can immediately notice the lower outer membrane permeability of *P. aeruginosa* when compared to that of *E. coli*.

**TABLE 5:** Permeability coefficients determined in *P. aeruginosa* PAO1 for a set of different β-lactams, belonging to penicillin, cephalosporin (1st, 2nd, and 3rd generation), and carbapenem families^*a*^

Permeability coefficients (nm/sec)		
Antibiotic	*P. aeruginosa* PAO1	*E. coli*
Ampicillin	0.008 ± 0.004	28 (70)
Benzylpenicillin	0.006 ± 0.002	
Cephaloridine	0.03 ± 0.01	
Cefoxitin	0.002 ± 0.0006	
Cefuroxime	0.001 ± 0.0004	
Cefotaxime	0.001 ± 0.0005	180 (72)
Imipenem	20 ± 9	1800 (71)
Meropenem	0.06 ± 0.01	300 (71)
Ertapenem	0.06 ± 0.02	
Doripenem	0.56 ± 0.38	
Biapenem	4.7 ± 1.4	

^*a*^ Values reported in the literature for *E. coli* are shown for comparison.

We then selected a penicillin (ampicillin), a first-generation cephalosporin (cephaloridine) and five carbapenems (imipenem, meropenem, ertapenem, doripenem, and biapenem) and determined their permeability coefficients in different mutant strains (Table 6). As expected, decreased values for imipenem uptake were detected in all the strains where OprD was deleted or mutated. The OprD mutants exhibited a 130-fold reduction in the permeability coefficient when compared to PAO1. The values obtained for the TNP004 mutant are similar to those for ARC5990 (PAO1*ΔoprD*), confirming that the porin is not expressed in TNP004.

**TABLE 6:** Permeability coefficients for different β-lactams determined for *P. aeruginosa* PAO1 and other porin(s) or efflux pump mutants^*a*^

Permeability coefficients (nm/sec)						
Antibiotic	PAO1	TNP004	ARC5990	ARC5170	ARC5782	ARC5998
Relevant characteristics		↓OprD	*ΔoprD*	*ΔopdP*	*ΔoprD, ΔopdP*	*ΔoprD, ΔopdP, ΔopdB, ΔopdC,* and *ΔopdT*
Ampicillin	0.008 ± 0.005	0.008 ± 0.003	0.02 ± 0.01	0.01 ± 0.002	0.01 ± 0.002	0.02 ± 0.005
Cephaloridine	0.03 ± 0.02	0.02 ± 0.004	ND	0.03 ± 0.01	0.03 ± 0.01	0.04 ± 0.01
Imipenem	20 ± 9	0.13 ± 0.07	0.14 ± 0.07	15 ± 5.8	0.13 ± 0.05	0.12 ± 0.06
Meropenem	0.06 ± 0.01	0.03 ± 0.02	0.03 ± 0.01	0.1 ± 0.05	0.006 ± 0.002	0.01 ± 0.005
Ertapenem	0.06 ± 0.02	0.03 ± 0.01	0.04 ± 0.02	0.02 ± 0.01	0.02 ± 0.01	0.02 ± 0.01
Doripenem	0.51 ± 0.35	0.16 ± 0.06	0.07 ± 0.03	0.11 ± 0.02	0.14 ± 0.11	0.11 ± 0.05
Biapenem	4.7 ± 1.4	3.4 ± 1.9	4.2 ± 2.7	7.2 ± 2.9	0.21 ± 0.13	0.12 ± 0.03

^*a*^ ND refers to a coefficient not determined.

This result is in accordance with the specific role of OprD in imipenem uptake, due to the structural identity between the C2 side-chain of imipenem and that of arginine, the natural substrate of the porin. For the other tested carbapenems, the increase in MIC values does not clearly map into a marked permeability decrease since we only measured a twofold difference between the single OprD mutants and the wild-type.

The permeability coefficients of ARC5170 (PAO1*ΔopdP*) were similar to those of the reference strain PAO1, suggesting that OpdP was not involved in antibiotic uptake.

Interestingly, a different result was noticed in the analysis of the mutant ARC5782, that lacks both OprD and OpdP. The imipenem permeability coefficient was similar to that of ARC5990 (PAO1*ΔoprD*), while, for meropenem and biapenem, the permeability coefficients were respectively tenfold and 30-fold lower than that of the wild-type PAO1.

These data underline a synergistic role of the OprD and OpdP porins in meropenem and biapenem uptake. An involvement of OpdP in meropenem permeation had already been suggested in the literature (24), but the diffusion of biapenem through this porin had never been described before.

We performed the same analysis on ARC5998 and obtained data similar to those observed with the double OprD and OpdP mutants, indicating that OpdB, OpdC, and OpdT are not primarily involved in the uptake of the tested antibiotics.

This approach allowed the evaluation of the specific permeability coefficients for various antibiotics in a series of *P. aeruginosa* strains (Table 6), which exhibit different levels of porin expressions.

We also highlighted the presence of compensatory effects in the permeation of antibiotics, in particular, the contribution of both OprD and OpdP porins in the internalization of meropenem and biapenem. Deleting each of these porins individually does not alter the entry rate of these antibiotics in the respective mutants, while in the mutant deprived of both porins, a marked decrease in permeability for both antibiotics is observed (tenfold and 30-fold, respectively). This outcome could not have been inferred from the MIC values, which are in fact identical in all mutants for the antibiotics mentioned above (Table 1).

The method described here can, in principle, be extended to other antibiotics and also to other gram-negative pathogens of clinical interest, representing a useful tool to study porin’s functionality.

### qRT-PCR

The relative expression of *oprD*, together with four other porins (*opdP*, *opdB*, *opdC*, and *opdT*) at four different time points of cellular growth was quantified by qRT-PCR. Total mRNAs were so extracted at OD_600_ of 0.6, 1.2, 1.6, and 2.0, corresponding, respectively, to the early, mid and late exponential, and early stationary phase.

*PA3340*, *gyrA,* and *cysG* genes, due to their relative stable expressions, were chosen as reference genes, and the exact protocol that results in their selection is reported in the Supplementary Material.

**Fig 4:**
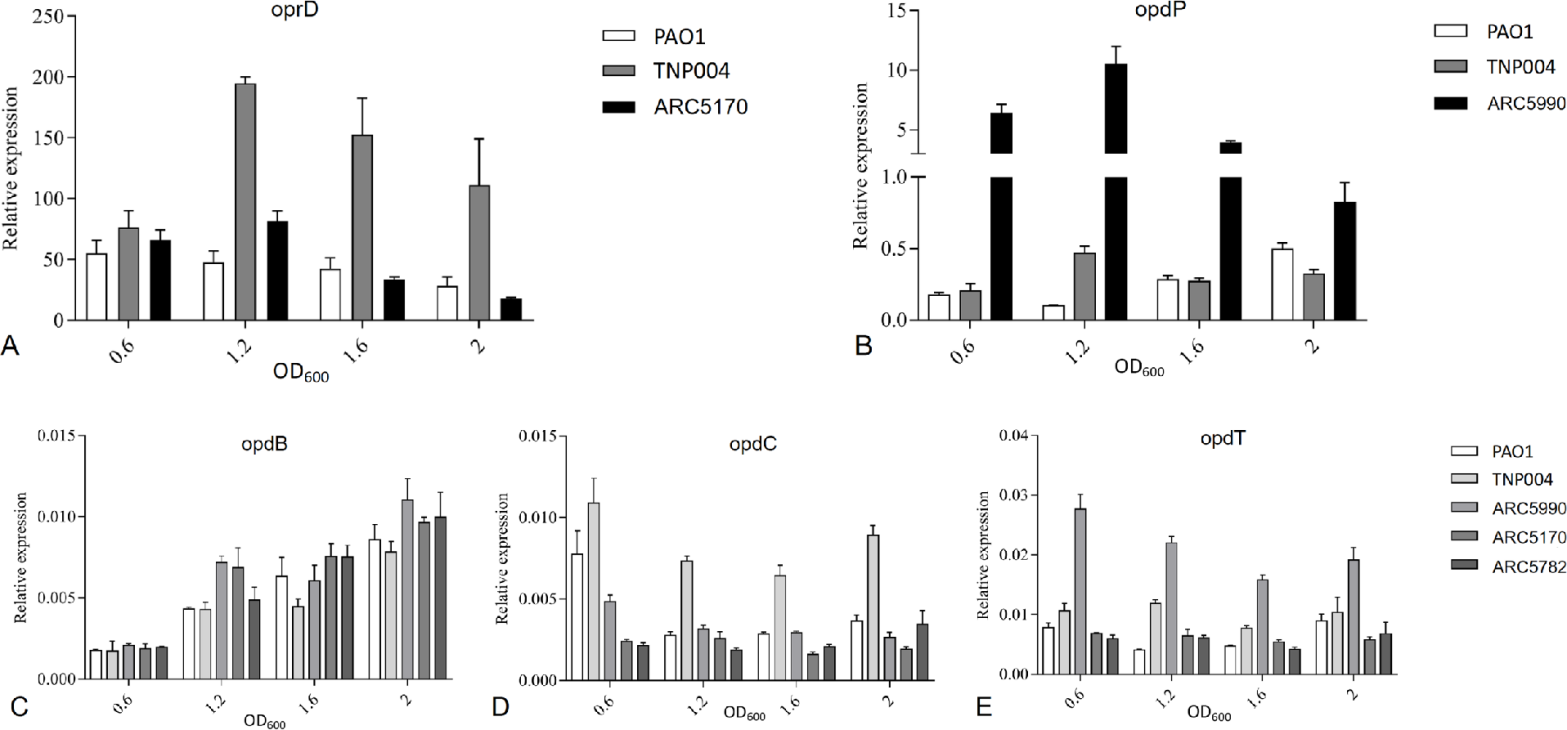
Relative expressions of (A) oprD, (B) opdP, (C) opdB, (D) opdC, and (E) opdT mRNAs in *P. aeruginosa* PAOI and four porin(s) mutant strains; mRNAs were extracted at four different points of bacterial growth, reported in the x-axis as the absorbance at 600 nm, and the relative expression reported on the y-axis is the mean of the transcription of three indipendent reference genes (PA3340, gyrA, and cysG). Data were analyzed by two-way analysis of variance (ANOVA), followed by Bonferroni multiple comparison *post-test*.

The relative *oprD* expression in *P. aeruginosa* PAO1, TNP004, and ARC5170 (PAO1*ΔopdP*) is reported in Fig. 4A. We confirmed that, as previously assumed, the expression of *oprD* mRNA is inversely proportional to cell density (53); as a consequence, the decreased expression of *oprD* observed at OD_600_ 1.6, compared to the early exponential phase (OD_600_ 0.6), might explain the differences found between carbapenem MICs and the permeability coefficients obtained in this study.

During our permeability measurements performed during the late exponential growth phase, as a consequence of the low physiological expression of OprD, the deletion of this porin does not produce a strong decrease in permeability for carbapenems, except for imipenem. In contrast, the MICs, whose determination involves the passage through the exponential phase in the overnight culture, show the effect of OprD’s higher expression in the early growth phase.

Interestingly, in TNP004, *oprD* mRNA appeared to be upregulated, but this did not reflect in the insertion of a functional porin in the outer membrane, as demonstrated by Western blot analysis. The bacteria are, therefore, able to respond to the lack of the functional porin by overexpressing *oprD* mRNA transcription, suggesting that the expression of this porin is controlled by a precise regulatory mechanism.

We performed the same screening on *opdP* mRNA in *P. aeruginosa* PAO1, TNP004, and ARC5990 (PAO1*ΔoprD*), and the results are shown in Fig. 4B.

In *P. aeruginosa* PAO1 the expression of OpdP does not seem to be regulated by cell density, but, in contrast to OprD, its expression is slightly increased during the early stationary phase. Moreover, its expression is increased 20-fold when *oprD* mRNA is not expressed as in the case of ARC5990 (PAO1*ΔoprD*). In TNP004, in fact, the relative *opdP* expression remains similar to that in PAO1, probably due to the simultaneous over-transcription of *oprD* mRNA. This finding highlights how the absence of a porin can be compensated for, but it does not fully elucidate the mechanism that prevails in the TNP004 strain.

It is important to notice that the relative mRNA expression of *opdP* in ARC5990 (PAO1*ΔoprD*) is ten times less than that of *oprD* in ARC5170 (PAO1*ΔopdP*); nevertheless, the permeability coefficients for all carbapenems, except for imipenem, are similar in the two considered strains.

However, OpdP has been described to exhibit a 30-fold higher conductance than OprD (30), and this can explain how a lower expression of this porin allows the uptake of all carbapenems at the same rate, with the exception of imipenem.

We finally quantified the relative mRNA expression of the *oprB, oprC,* and *oprT* porin genes, previously chosen for their high relative expression in minimal medium (24), in *P. aeruginosa* PAO1, TNP004, ARC5990 (PAO1*ΔoprD*), ARC5170 (PAO1*ΔopdP*), and ARC5782 (PAO1*ΔoprD, ΔopdP*).

The results for these porins are reported in Fig. 4C through E, respectively, and show that all three porins have a basal expression level, while only that of *opdB* mRNA seems to be directly proportional to cell density. However, their low expression does not seem to be crucial for antibiotic uptake, as shown by the MICs and the permeability coefficient determinations.

We also quantified the relative expression of *oprD* mRNA in two strains selected during the multistep resistance experiment. We chose LG01, derived from *P. aeruginosa* PAO1, carrying a mutated *oprD* sequence, and LG03, derived from ARC5170 (PAO1*ΔopdP*) that possesses an intact *oprD,* and we compared the relative expression of the porin at A_600_ = 1.6, using the reference genes mentioned above.

**Fig 5:**
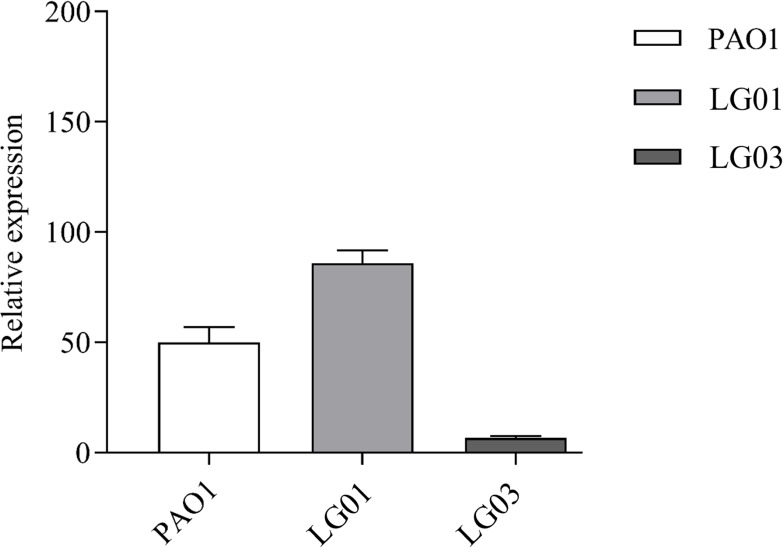
Relative expression of oprD mRNA in *P. aeruginosa* PAOI, LG01, and LG03; mRNAS were extracted at A600 = 1.6, and the relative expression reported on the y axis is the mean of the transcriptions of three indipendent reference genes (PA3340, gyrA, and cysG) mentioned above. Data were analyzed by two-way analysis of variance (ANOVA), followed by Bonferroni multiple comparison *post-test*.

The results reported in Fig. 5 show an increased expression of *oprD* mRNA, similarly to TNP004, in LG01 when compared to the wild-type, most likely due to the synthesis of a mutated unstable porin.

Interestingly, *oprD* mRNA expression in LG03 appears to be tenfold downregulated compared to PAO1, probably as a consequence of the already mentioned mutations found in the *nalD* or *dsbS* genes.

Thus, the so far undocumented mechanism results in a downregulation of the porin (verified by qRT-PCR and Western blot), which causes carbapenem resistance highlighted by the MIC values.

These experiments shed further light on OpdP, demonstrating that this porin is less expressed during the exponential growth phase, while its production increases when the bacterial culture enters the latent phase. This phenomenon turns out to be of particular importance during the permeability determinations, which were performed with cultures in the late exponential growth phase, but also during an *in vivo* infection. Conversely, the production of OprD follows an inverse pathway (Fig. 5A and B), and this mechanism contributes to explain why the MIC values appear to be mostly influenced by OprD. The determination of the MICs necessarily requires an exponential growth phase and, therefore, more affected by the presence/absence of the OprD porin in *P. aeruginosa*.

Besides this experimental evidence, it is, therefore, important to highlight how the simultaneous absence of OprD and OpdP porins might determine the poor efficacy of meropenem and biapenem during an antibiotic therapy, while the single deletion of OpdP can favor the appearance of a carbapenem resistance phenotype after exposure to meropenem in a concentration close to the MIC value.

Therefore, the presence of mutations/deletions of the OpdP porin should receive greater consideration from a clinical point of view, and further studies on the expression of this porin in clinical strains could lead to a better understanding of the resistance mechanisms mediated by this porin.

## MATERIALS AND METHODS

### Bacterial strains, growth conditions, plasmids, and antibiotics

Antibiotics were purchased from Sigma-Aldrich and nitrocefin from Oxoid Ltd. (Basingstoke, UK).

Biapenem was kindly provided by Dr. O. Lomovskaya from The Medicines Company (San Diego, CA, USA).

Bacterial strains are listed in Table 7, and the porin(s) deletion assessment is reported in supplemental data. Plasmids are reported in Table S1. Bacteria were grown aerobically at 37°C in LB, purchased from Sigma-Aldrich.

**TABLE 7:** Collection of *P. aeruginosa* strains used in the study^*a*^

*P. aeruginosa*	Relevant characteristics	Reference
PAO1	Wild-type	BCCM
PAO1-Jap	Presumptive TNP004 parental strain	(47)
TNP004	**↓**OprD	(47)
ARC545	ARC presumptive parental strain	(24)
ARC5990	*ΔoprD*	(24)
ARC5170	*ΔopdP*	(24)
ARC5782	*ΔoprD*, *ΔopdP*	(24)
ARC5998	*ΔoprD*, *ΔopdC*, *ΔopdP*, *ΔopdT*, and *ΔopdB*	(24)
LG01	*oprD* mutant, derived from PAO1	This study
LG02	*oprD* mutant, derived from ARC5170	This study
LG03	*oprD* mutant, derived from ARC5170	This study
LG04	*oprD* mutant, derived from ARC5170	This study
LG05	*oprD* mutant, derived from ARC5170	This study
LG06	*oprD* mutant, derived from ARC5170	This study
LG07	*oprD* mutant, derived from ARC5170	This study

^*a*^ **↓**OprD indicates a downregulated expression of OprD.

To perform planktonic cultures, a single colony was inoculated into liquid medium and incubated overnight (14–16 hours). Cultures were then diluted 1:20 into fresh media, and bacterial growth was monitored by following the absorbance at 600 nm using an Ultrospec 10 spectrophotometer (BioChrom, St Albans, UK).

The pKT240blaR shuttle vector (45) includes the *B. licheniformis blaR-CTD* gene under the control of the *lpp-lac* fusion promoter. The pKT240neg vector was derived from pKT240blaR where *blaR-CTD* was excised. Finally, pKT240blaR-gen was obtained by the addition of the *aac1* gene, in order to confer resistance to gentamicin. Both latter constructs were produced using the Gibson assembly technology (63), by amplifying fragments of the pKT240blaR plasmid and the *aac1* gene in the case of pKT240blaR-gen. Primers are listed in Table S2. The different plasmids were transformed in *E. coli* DH5α-Proteobacterium *Pseudomonas aeruginosa*. They were purified using the NucleoBond Xtra Maxi kit (Macharey-Nagel, Bethlehem, PA) and used to transform *P. aeruginosa* strains by electroporation using the Gene Pulser Xcell Electroporation System (Bio-Rad, Hercules, CA, USA), in an ice-cold 0.2 cm cuvette with the following parameters: 200 Ω, 25 µF, and 2.5 kV.

We further selected new *P. aeruginosa* mutant strains where the permeability of the outer membrane is affected. Those strains were obtained with the help of a multistep resistance experiment. Briefly, *P. aeruginosa* PAO1 and *P. aeruginosa* ARC5170 (PAO1*ΔopdP*), grown in liquid LB medium, were harvested in the mid- and late-exponential growth phase (A_600_ ~1.2 and 1.6) and streaked (10^9^ CFU) onto Petri plates containing two different meropenem concentrations (1 and 2 µg/mL), higher than the MICs determined for both parental strains (0.5 µg/mL). The plates were incubated for 16 hours at 37°C. Seven colonies (*P. aeruginosa* LG01, derived from *P. aeruginosa* PAO1, and *P. aeruginosa* LG02-LG07 derived from *P. aeruginosa* ARC5170) were then randomly selected for further characterizations.

### Susceptibility testing

Antimicrobial susceptibility was evaluated by broth microdilutions in cation-adjusted Mueller–Hinton broth (MHBII), purchased from Sigma-Aldrich, according to the Clinical and Laboratory Standards Institute guidelines (46). *P. aeruginosa* ATCC 27853 was used as a control strain, and data were collected in triplicate independent experiences.

### PCR amplification, DNA sequencing, and whole-genome sequencing (wgs)

PCR screenings on different targets were performed on crude extracts using OneTaq polymerase (New England Biolabs, Ipswich, MA, USA), while Q5 High-Fidelity enzyme (New England Biolabs) was used in case of a successive Sanger sequencing analysis.

The *oprD* sequence was determined by Sanger sequencing of PCR products, obtained using the pair of primers oprD_flankF and oprD_flankR (Table S2).

The nucleotide and protein sequences were analyzed using the blastn and blastp algorithms, available on the National Center of Biotechnology Information website (http://www.ncbi.nlm.nih.gov), and the alignments of the translated amino acid sequences of OprD were performed using the software Alignx (InforMax, Bethesda, MD, USA).

To perform whole-genome sequencing, genomic DNA was extracted using the NucleoSpin DNA Plus kit (Macherey-Nagel). Samples were then processed on a NovaSeq (Illumina, Inc., San Diego, CA, USA) sequencer, generating paired-end reads (2 × 150); raw reads were corrected by a homemade workflow, performing various steps of analysis, using software included in the BBTools package (64); briefly, reads were overlapped with BBMerge and subsequently quality-trimmed, and any remaining adapters were removed by the BBduk function. Tadpole and BBMap were in sequence used to perform a quick assembly, and BBduk was used for quality calibration; finally, BBNorm was used to normalize the coverage, and Tadpole was used for a final process of error-correction.

The sequence mapping was carried out with the Geneious (v10.2.6) software (65). Genome assembly was achieved by mapping single verified reads to a reference *P. aeruginosa* genome sequence (strain PAO1, GenBank accession number AE004091), and the resulting variations have been applied to the reference to generate the strain sequences.

Both Sanger and whole-genome sequencing were carried out at the GIGA-Genomics platform (GIGA-Genomics, Liège, Belgium).

### RNA extraction, cDNA synthesis, and quantitative qRT-PCR

Quantitative real-time PCR (qRT-PCR) was used to compare the expressions of porins genes, at four different moments of cellular growth (A_600_ = 0.6, 1.2, 1.6, and 2.0).

Total RNA isolation of the different aliquots was performed using the NucleoSpin RNA Plus kit (Macherey-Nagel), and rDNase (Macherey-Nagel) digestion was subsequently performed to eliminate any DNA trace. The samples were finally purified using the NucleoSpin RNA Clean-up XS kit (Macherey-Nagel) according to the manufacturer’s recommendations.

RNA quantification was performed by measuring the absorbance at 260 nm with the help of a NanoVue spectrophotometer (GE Healthcare, Little Chalfont, UK). Then, 1 µg of RNA was retrotranscribed using the SuperScript III reverse transcriptase (Invitrogen, Waltham, MA, USA), triggered by random hexamers and supplemented with 0.4 U/µL of Ribosafe RNase Inhibitor (Bioline, USA). The reaction was carried out at 25°C for 5 minutes, 50°C for 60 minutes, and stopped by heating to 70°C for 15 minutes. The newly synthesized cDNA was diluted to 1:50 in RNase/DNase-free water and used as a target for qRT-PCR.

Amplifications were performed in 384-well plates with a QuantStudio 5 Real-Time PCR system (Thermo Fisher Scientific, Waltham, MA, USA) using Takyon Low Rox SYBR MasterMix Eurogentec (Seraing, Belgium); the list of primers used is reported in Table S3.

A control reaction was performed for each sample by using the original RNA mixture to verify the absence of residual DNA. Experiments were reproduced in four biological replicates and three technical replicates for each target gene.

The quality of the quantitative PCR was checked by the analysis of dissociation and amplification curves. For each primer pair, the mean reaction efficiencies were calculated using the LinRegPCR software (66) (Table S3). Those values were used to quantify relative gene expression levels by normalization using three reference genes (*PA3340*, *gyrA,* and *cysG*) with the qBase software (Biogazelle) (67). The reference genes were chosen because they were similarly expressed in the various growth phases. The adequacy of the reference genes to normalize gene expression in the experimental conditions was checked using the geNorm module in qBase (68).

### BlaR-CTD affinity

BlaR-CTD is the soluble C-terminal domain of the BlaR transmembrane protein that displays a high affinity for β-lactams characterized by the acylation constant (*k*_2_/K′), the second-order rate constant for the formation of the acyl-enzyme adduct characterizing the acylation step efficiency. This rate constant can be determined by incubating a known concentration of the antibiotic, whose k_2_/K′ is to be measured, together with a known concentration of a reporter antibiotic (r), whose kinetic parameters are known. The proportion of each acyl-enzyme formed at saturation depends on the concentration and k_2_/K′ value of each antibiotic (equation 1) (69). Our reporter molecule was nitrocefin (Δε^482^ = 15,000 M^−1^ cm^−1^).

The assay can be described as follows:


(1) $$\begin{array}{l} \frac{[EC^{*}]r}{\left[EC^{*}\right]}=\frac{\left(\frac{k_{2}}{K^{\prime }}\right)r*[C]r}{\left(\frac{k_{2}}{K^{\prime }}\right)*[C]}\;and\;[EC^{*}]r+[EC^{*}]=[E_{0}]\\ \frac{[EC{]}_{r}}{[E_{0}]-[EC{]}_{r}}=\frac{(k_{2}/K^{\prime }{)}_{r}[C{]}_{r}}{\left(k_{2}/K^{\prime })[C\right]} \end{array},$$

where [EC*]r and [EC*] are the concentrations of BlaR-nitrocefin and BlaR-antibiotic acyl-enzymes, respectively. [C]r and [C] correspond to the concentrations of nitrocefin and of the tested antibiotic, respectively. (*k_2_/K’*)r and (*k_2_/K’*) represent the acylation rate constants of BlaR-CTD for the nitrocefin and the tested antibiotic.

We first determined the acylation rate constant of BlaR-CTD for nitrocefin. Purified BlaR-CTD was previously purified at the CIP (70). BlaR-CTD (20 µM), was added to a solution of nitrocefin (80 µM) containing increasing concentrations (25–600 µM) of ampicillin, whose k_2_/K′ is known (1.3 10^6^ M^−1^· s^−1^) (70). The variation of A_482_ is directly proportional to the concentration of the BlaR-CTD-nitrocefin adduct. The k_2_/K′ for nitrocefin was then calculated (3.6 10^6^ M^−1^· s^−1^) and used as a competitor to determine the other affinity values. The acylation rate constants were so determined for cefalotin, ceftazidime, imipenem, meropenem, ertapenem, biapenem, and doripenem.

### β-Lactamase assays

The production of the class C AmpC β-lactamases was measured in crude cell extracts from the different *P. aeruginosa* PAO1 cultures as follows. The growth of the bacteria in LB at 37°C was monitored by measuring A_600_. At a value of 1.6, the culture was divided into two 10 mL aliquots. One aliquot was used as a control. Antibiotic was added to the second aliquot at a final concentration equal to the maximum concentration of the antibiotic tested in the permeability assay (Table 8). The cultures were then incubated for a time corresponding to the incubation time with the selected antibiotic in the permeability test. One mL of each culture was then centrifuged at 13,000 *g* for 10 minutes. The pellet was washed twice and resuspended in 1 mL of 10 mM PBS buffer pH 7.4. Cells were lysed by sonication with the Bioruptor Plus Diagenode (Seraing, Belgium). The cellular extract was clarified by centrifugation at 13,000 *g* for 30 minutes at 4°C.

**TABLE 8:** Different antibiotics concentrations tested during permeability experiments

Antibiotics	Concentrations tested (μM)		
Benzylpenicillin	40	20	10
Cefoxitin	30	15	7.5
Cefuroxime	60	30	15
Cefotaxime	60	30	15
Ampicillin	20	10	5
Cephaloridine	8	4	2
Imipenem	0.02	0.01	0.005
Imipenem^*a*^	4	2	1
Meropenem	8	4	2
Meropenem^*b*^	20	10	5
Ertapenem	7.5	5	2.5
Doripenem	4	2	1
Biapenem	0.04	0.02	0.01
Biapenem^*b*^	4	2	1

^*a*^ * refers to tests performed on strains lacking the OprD porin.

^*b*^ while ^ refers to tests performed on strains deprived of both OprD and OpdP porins.

Positive controls for AmpC induction in *P. aeruginosa* PAO1 were analysed as previously described and incubated for six hours in presence of 50 µM ampicillin and 50 µM cefoxitin. The negative controls were made by the culture without antibiotic, grown for the different tested incubation times.

The protein concentration in each extract was measured with the help of a BCA protein assay kit (Pierce, Rockford, IL). The β-lactamase activity of the extract was determined by measuring the initial rate of hydrolysis of 100 µM nitrocefin. All the enzymatic assays were performed in 10 mM PBS buffer pH 7.4 at 30°C. The specific activity of the different samples was the rate of hydrolysis of each substrate expressed in nmoles per minute per milligram of protein.

### Permeability determination

The antibiotic flux passing through the outer membrane can be described by the Flick’s first law of flux (2).


(2) $$J=-D\cdot \;A\cdot \frac{\Delta C}{\Delta x},$$

where J is the flux of the antibiotic through the outer membrane OM, D the diffusion coefficient of the antibiotic, A the OM area (assumed as 132 cm^2^), ΔC the concentration gradient of the antibiotic, and Δx the OM thickness.

β-lactam flux in *P. aeruginosa* can be characterized by the permeability coefficient P (3), that is defined as the ratio between the diffusion coefficient and the OM thickness. It can be defined as:


(3) $$P=-\frac{D}{\Delta x}.$$

The antibiotic flux is defined by equation 4:


(4) J=P⋅A⋅ΔC.

**Fig 6:**
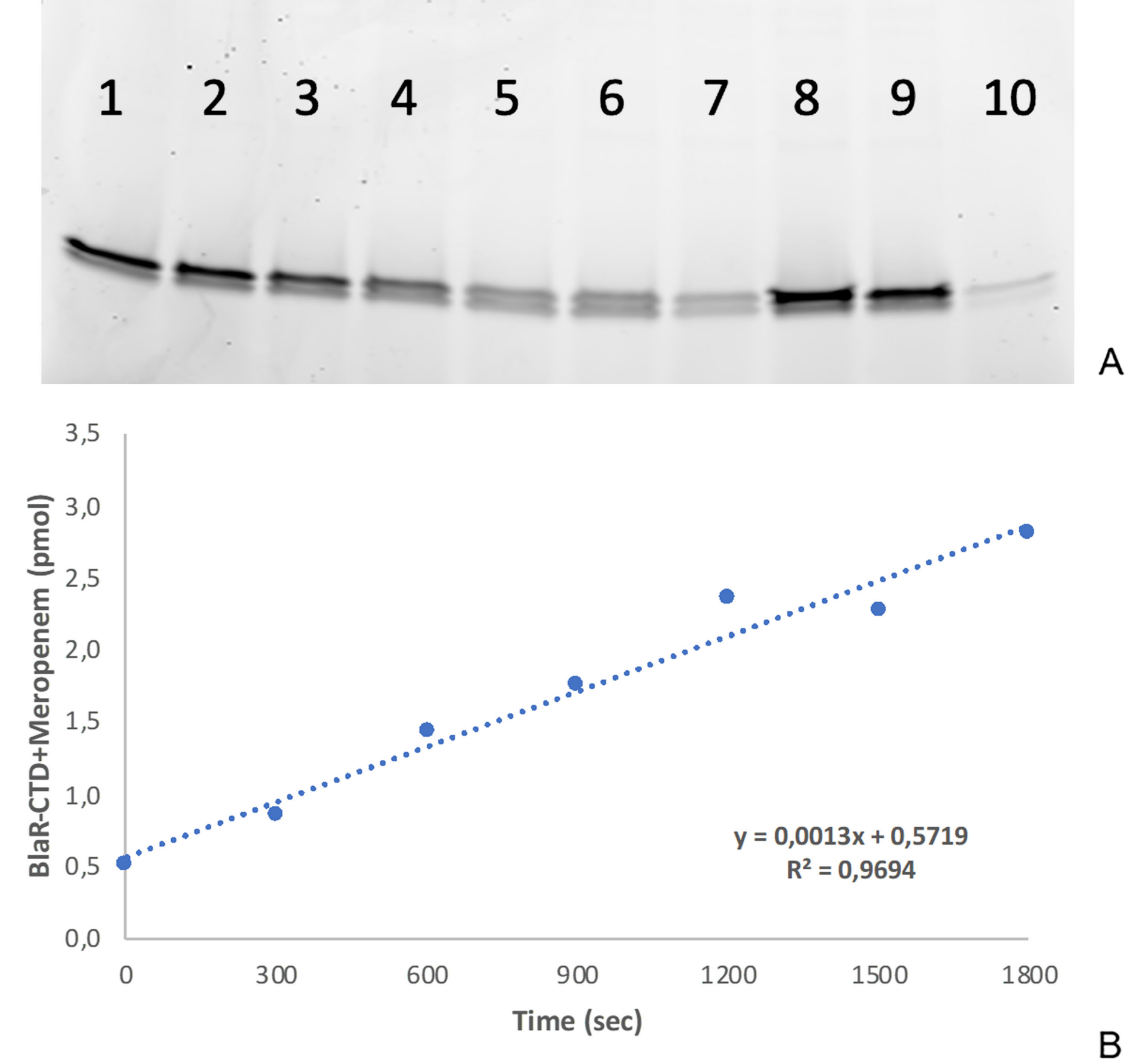
(A) Densitometric analysis of the fluorescence signal of the BlaR-CTD-Bocillin adduct, shown as an example; samples from 1 to 7 represent different aliquots, taken every 5 minutes for determining meropenem uptake in ARC5782 (PAO1ΔoprD, ΔopdP); samples 8 and 9 are aliquots taken before the addition of meropenem to the culture, representing the total quantity of produced BlaR-CTD; sample 10 is a not sonicated aliquot, used to quantify possible BlaR-CTD release in the medium. (B) Graph representing the increase of the meropenem-BlaR-CTD adduct as a function of time; this quantification was made possible by subtracting the values obtained during the experiment (samples 1-7 of panel A) from the total BlaR-CTD produced in an aliquot (samples 8 or 9 of panel A). The slope of the line represents the antibiotic flux passing through the outer membran.

The estimation of the antibiotic flux can be achieved by expressing the high affinity BlaR-CTD in the bacterial periplasm, allowing the direct quantification of the β-lactam concentration present in the periplasmic space, and consequently to measure the permeability coefficient (5)


(5) $$P=\frac{d(EC^{*})/dt}{A\cdot [C_{e}]}$$

where EC* is the concentration of the BlaR-CTD-β-lactam adduct and [C_e_] the external β-lactam concentration.

d(EC*)/dt is equal to the slope of the line reflecting the increase of the acyl-enzyme concentration *vs* the incubation time (Fig. 6B).

Antibiotic diffusion in the periplasmic space was analyzed in planktonic cultures. The different *P. aeruginosa* strains, previously transformed with pKT240blaR (or pKT40blaR-gen in the case of TNP004), were grown in LB medium added with selection antibiotic (50 µg/mL tetracycline or 10 µg/mL gentamicin, respectively). When the culture reached the late exponential phase (A_600_ ≃ 1.6) a prefixed β-lactam concentration was added to the medium and aliquots (1 mL) were harvested at different incubation times. Two microliters of the metallo-β-lactamase VIM-2 (1 mg/mL) was added to the bacterial culture to hydrolyze all the antibiotics present outside the cells and consequently interrupt its permeation in an active form. EDTA (1 mM) was then added in order to chelate the metal ions and inactivate the metallo-β-lactamase. The crude extract was obtained by means of ten cycles of refrigerated sonication at 4°C, performed with the Bioruptor Plus Diagenode. The quantification of BlaR-CTD acylated by the tested antibiotic was performed by adding 2.5 µM Bocillin FL (Invitrogen), a fluorescent derivative of penicillin V, to the crude extracts. The proteins of the different cell extracts were separated by SDS-PAGE, and the fluorescence intensity of BlaR-CTD recorded using a Typhoon Trio +imager and Image Quant TL software (GE Healthcare) (Fig. 6A). The quantification of the BlaR-CTD-Bocillin adduct *vs* the incubation time in presence of an unlabeled β-lactam allowed the determination of the quantity of BlaR-CTD acylated with the unlabelled compound. Analyses were performed in duplicate at three different antibiotic concentrations as reported in Table 8.

### Protein extraction, SDS, and Western Blot

The outer membrane profiling was performed on LB cultures as previously described (71).

SDS-PAGE was carried out according to the Laemmli protocol, using 4%–20% Mini-Protean (Bio-Rad) gels and Coomassie blue staining.

For Western blot analysis, proteins were electroblotted onto a PVDF membrane, using a commercial kit (Bio-Rad), and the protein marker V (pre-stained) VWR (Radnor, PA, USA) was used.

The membranes were then incubated with 1:2,000 rabbit-derived anti-OprD polyclonal antibody (72), kindly provided us by Dr Thilo Köhler (University of Geneva, Switzerland) and finally with the secondary antibody 1:5,000 goat anti-rabbit HRP antibody (Bio-Rad).

For the revelation, Clarity Western ECL substrate kit (Bio-Rad) was added, and signals were detected using an ImageQuant LAS 4000 camera (GE Healthcare).

## Supplementary Material

Reviewer comments

## Data Availability

The assembled genomic sequences of P. aeruginosa isolates were deposited under the Bioproject number PRJNA985251 in the NCBI database (https://www.ncbi.nlm.nih.gov/bioproject/) Sanger sequencing data have been deposited on Genbank, under the accession numbers OR069747, OR069748, OR069749 and OR069750.

## SUPPLEMENTAL MATERIAL

Fig. S1 spectrum.00495-24-s0001.tiff Supplemental figure 1.

Fig. S2 spectrum.00495-24-s0002.tiff Supplemental figure 2.

Fig. S3 spectrum.00495-24-s0003.tiff Supplemental figure 3.

Supplemental material spectrum.00495-24-s0004.docx Porin(s) deletion assessment; RT-PCR reference genes.

Table S1 spectrum.00495-24-s0005.docx Collection of plasmids used in this study.

Table S2 spectrum.00495-24-s0006.docx Collection of primers used in this study for PCR amplification and sequencing.

Table S3 spectrum.00495-24-s0007.docx Sequences and reaction efficiencies of quantitative RT-PCR primer pairs.

OPEN PEER REVIEW reviewer-comments.pdf An accounting of the reviewer comments and feedback.

ASM does not own the copyrights to Supplemental Material that may be linked to, or accessed through, an article. The authors have granted ASM a non-exclusive, world-wide license to publish the Supplemental Material files. Please contact the corresponding author directly for reuse.

## References

[1] Weber DJ, Rutala WA, Sickbert-Bennett EE, Samsa GP, Brown V, Niederman MS. 2007. Microbiology of ventilator-associated pneumonia compared with that of hospital-acquired pneumonia. Infect Control Hosp Epidemiol 28:825–831. doi:10.1086/518460 17564985

[2] Lamas Ferreiro JL, Álvarez Otero J, González González L, Novoa Lamazares L, Arca Blanco A, Bermúdez Sanjurjo JR, Rodríguez Conde I, Fernández Soneira M, de la Fuente Aguado J. 2017. Pseudomonas aeruginosa urinary tract infections in hospitalized patients: mortality and prognostic factors. PLoS One 12:e0178178. doi:10.1371/journal.pone.0178178 28552972 PMC5446154

[3] Jurado-Martín I, Sainz-Mejías M, McClean S. 2021. Pseudomonas aeruginosa: an audacious pathogen with an adaptable Arsenal of virulence factors. Int J Mol Sci 22:3128. doi:10.3390/ijms22063128 33803907 PMC8003266

[4] Staudinger BJ, Muller JF, Halldórsson S, Boles B, Angermeyer A, Nguyen D, Rosen H, Baldursson O, Gottfreðsson M, Guðmundsson GH, Singh PK. 2014. Conditions associated with the cystic fibrosis defect promote chronic Pseudomonas aeruginosa infection. Am J Respir Crit Care Med 189:812–824. doi:10.1164/rccm.201312-2142OC 24467627 PMC4225830

[5] Angrill N, Gallego M, Font J, Vallés J, Morón A, Monsó E, Rello J. 2020. Determinants of empirical antipseudomonal antibiotic prescription for adults with pneumonia in the emergency department. BMC Pulm Med 20:83. doi:10.1186/s12890-020-1115-0 32245452 PMC7126131

[6] Curran CS, Bolig T, Torabi-Parizi P. 2018. Mechanisms and targeted therapies for Pseudomonas aeruginosa lung infection. Am J Respir Crit Care Med 197:708–727. doi:10.1164/rccm.201705-1043SO 29087211 PMC5855068

[7] Zhanel GG, Lawrence CK, Adam H, Schweizer F, Zelenitsky S, Zhanel M, Lagacé-Wiens PRS, Walkty A, Denisuik A, Golden A, Gin AS, Hoban DJ, Lynch JP III, Karlowsky JA. 2018. Imipenem-relebactam and meropenem-vaborbactam: two novel carbapenem-β-lactamase inhibitor combinations. Drugs (Abingdon Engl) 78:65–98. doi:10.1007/s40265-017-0851-9 29230684

[8] Sader HS, Castanheira M, Duncan LR, Mendes RE. 2021. Antimicrobial activities of ceftazidime/avibactam, ceftolozane/tazobactam, imipenem/relebactam, meropenem/vaborbactam, and comparators against Pseudomonas aeruginosa from patients with skin and soft tissue infections. Int J Infect Dis 113:279–281. doi:10.1016/j.ijid.2021.10.022 34670144

[9] Mesaros N, Nordmann P, Plésiat P, Roussel-Delvallez M, Van Eldere J, Glupczynski Y, Van Laethem Y, Jacobs F, Lebecque P, Malfroot A, Tulkens PM, Van Bambeke F. 2007. Pseudomonas aeruginosa: resistance and therapeutic options at the turn of the new millennium. Clin Microbiol Infect 13:560–578. doi:10.1111/j.1469-0691.2007.01681.x 17266725

[10] Campbell JI, Ciofu O, Høiby N. 1997. Pseudomonas aeruginosa isolates from patients with cystic fibrosis have different beta-lactamase expression phenotypes but are homogeneous in the ampC-ampR genetic region. Antimicrob Agents Chemother 41:1380–1384. doi:10.1128/AAC.41.6.1380 9174204 PMC163920

[11] Luzzaro F, Endimiani A, Docquier JD, Mugnaioli C, Bonsignori M, Amicosante G, Rossolini GM, Toniolo A. 2004. Prevalence and characterization of metallo-beta-lactamases in clinical isolates of Pseudomonas aeruginosa. Diagn Microbiol Infect Dis 48:131–135. doi:10.1016/j.diagmicrobio.2003.09.005 14972383

[12] Zavascki AP, Carvalhaes CG, Picão RC, Gales AC. 2010. Multidrug-resistant Pseudomonas aeruginosa and Acinetobacter baumannii: resistance mechanisms and implications for therapy. Expert Rev Anti Infect Ther 8:71–93. doi:10.1586/eri.09.108 20014903

[13] Hong DJ, Bae IK, Jang IH, Jeong SH, Kang HK, Lee K. 2015. Epidemiology and Characteristics of Metallo-β-Lactamase-Producing Pseudomonas aeruginosa. Infect Chemother 47:81–97. doi:10.3947/ic.2015.47.2.81 26157586 PMC4495280

[14] Sugawara E, Nestorovich EM, Bezrukov SM, Nikaido H. 2006. Pseudomonas aeruginosa porin OprF exists in two different conformations. J Biol Chem 281:16220–16229. doi:10.1074/jbc.M600680200 16595653 PMC2846725

[15] Chevalier S, Bouffartigues E, Bodilis J, Maillot O, Lesouhaitier O, Feuilloley MGJ, Orange N, Dufour A, Cornelis P. 2017. Structure, function and regulation of Pseudomonas aeruginosa porins. FEMS Microbiol Rev 41:698–722. doi:10.1093/femsre/fux020 28981745

[16] Nakae T, Nakajima A, Ono T, Saito K, Yoneyama H. 1999. Resistance to beta-lactam antibiotics in Pseudomonas aeruginosa due to interplay between the MexAB-OprM efflux pump and beta-lactamase. Antimicrob Agents Chemother 43:1301–1303. doi:10.1128/AAC.43.5.1301 10223959 PMC89266

[17] Li XZ, Plésiat P, Nikaido H. 2015. The challenge of efflux-mediated antibiotic resistance in Gram-negative bacteria. Clin Microbiol Rev 28:337–418. doi:10.1128/CMR.00117-14 25788514 PMC4402952

[18] Castanheira M, Doyle TB, Smith CJ, Mendes RE, Sader HS. 2019. Combination of MexAB-OprM overexpression and mutations in efflux regulators, PBPs and chaperone proteins is responsible for ceftazidime/avibactam resistance in Pseudomonas aeruginosa clinical isolates from US hospitals. J Antimicrob Chemother 74:2588–2595. doi:10.1093/jac/dkz243 31225882

[19] Trias J, Nikaido H. 1990. Outer membrane protein D2 catalyzes facilitated diffusion of carbapenems and penems through the outer membrane of Pseudomonas aeruginosa. Antimicrob Agents Chemother 34:52–57. doi:10.1128/AAC.34.1.52 2109575 PMC171519

[20] Lister PD, Wolter DJ, Hanson ND. 2009. Antibacterial-resistant Pseudomonas aeruginosa: clinical impact and complex regulation of chromosomally encoded resistance mechanisms. Clin Microbiol Rev 22:582–610. doi:10.1128/CMR.00040-09 19822890 PMC2772362

[21] Tamber S, Ochs MM, Hancock REW. 2006. Role of the novel OprD family of porins in nutrient uptake in Pseudomonas aeruginosa. J Bacteriol 188:45–54. doi:10.1128/JB.188.1.45-54.2006 16352820 PMC1317591

[22] Eren E, Vijayaraghavan J, Liu J, Cheneke BR, Touw DS, Lepore BW, Indic M, Movileanu L, van den Berg B. 2012. Substrate specificity within a family of outer membrane carboxylate channels. PLoS Biol 10:e1001242. doi:10.1371/journal.pbio.1001242 22272184 PMC3260308

[23] Tamber S, Hancock REW. 2006. Involvement of two related porins, OprD and OpdP, in the uptake of arginine by Pseudomonas aeruginosa. FEMS Microbiol Lett 260:23–29. doi:10.1111/j.1574-6968.2006.00293.x 16790014

[24] Isabella VM, Campbell AJ, Manchester J, Sylvester M, Nayar AS, Ferguson KE, Tommasi R, Miller AA. 2015. Toward the rational design of carbapenem uptake in Pseudomonas aeruginosa. Chem Biol 22:535–547. doi:10.1016/j.chembiol.2015.03.018 25910245

[25] Chalhoub H, Sáenz Y, Rodriguez-Villalobos H, Denis O, Kahl BC, Tulkens PM, Van Bambeke F. 2016. High-level resistance to meropenem in clinical isolates of Pseudomonas aeruginosa in the absence of carbapenemases: role of active efflux and porin alterations. Int J Antimicrob Agents 48:740–743. doi:10.1016/j.ijantimicag.2016.09.012 28128097

[26] Atrissi J, Milan A, Bressan R, Lucafò M, Petix V, Busetti M, Dolzani L, Lagatolla C. 2021. Interplay of OpdP porin and chromosomal carbapenemases in the determination of carbapenem resistance/susceptibility in Pseudomonas aeruginosa. Microbiol Spectr 9:e0118621. doi:10.1128/Spectrum.01186-21 34585948 PMC8557820

[27] Kiely PD, O’Callaghan J, Abbas A, O’Gara F. 2008. Genetic analysis of genes involved in dipeptide metabolism and cytotoxicity in Pseudomonas aeruginosa PAO1. Microbiology (Reading, Engl) 154:2209–2218. doi:10.1099/mic.0.2007/015032-0 18667554

[28] Pletzer D, Lafon C, Braun Y, Köhler T, Page MGP, Mourez M, Weingart H. 2014. High-throughput screening of dipeptide utilization mediated by the ABC transporter DppBCDF and its substrate-binding proteins DppA1-A5 in Pseudomonas aeruginosa. PLoS One 9:e111311. doi:10.1371/journal.pone.0111311 25338022 PMC4206461

[29] Asfahl KL, Walsh J, Gilbert K, Schuster M. 2015. Non-social adaptation defers a tragedy of the commons in Pseudomonas aeruginosa quorum sensing. ISME J 9:1734–1746. doi:10.1038/ismej.2014.259 25615439 PMC4511930

[30] Liu J, Wolfe AJ, Eren E, Vijayaraghavan J, Indic M, van den Berg B, Movileanu L. 2012. Cation selectivity is a conserved feature in the OccD subfamily of Pseudomonas aeruginosa. Biochim Biophys Acta 1818:2908–2916. doi:10.1016/j.bbamem.2012.07.009 22824298 PMC3424372

[31] Smalley NE, Schaefer AL, Asfahl KL, Perez C, Greenberg EP, Dandekar AA. 2022. Evolution of the quorum sensing regulon in cooperating populations of Pseudomonas aeruginosa. MBio 13:e0016122. doi:10.1128/mbio.00161-22 35294222 PMC8863103

[32] Ude J, Tripathi V, Buyck JM, Söderholm S, Cunrath O, Fanous J, Claudi B, Egli A, Schleberger C, Hiller S, Bumann D. 2021. Outer membrane permeability: antimicrobials and diverse nutrients bypass porins in Pseudomonas aeruginosa. Proc Natl Acad Sci U S A 118:e2107644118. doi:10.1073/pnas.2107644118 34326266 PMC8346889

[33] Zimmermann W. 1980. Penetration of beta-lactam antibiotics into their target enzymes in Pseudomonas aeruginosa: comparison of a highly sensitive mutant with its parent strain. Antimicrob Agents Chemother 18:94–100. doi:10.1128/AAC.18.1.94 6774666 PMC283945

[34] Yoshimura F, Nikaido H. 1982. Permeability of Pseudomonas aeruginosa outer membrane to hydrophilic solutes. J Bacteriol 152:636–642. doi:10.1128/jb.152.2.636-642.1982 6813310 PMC221510

[35] Livermore DM, Davy KW. 1991. Invalidity for Pseudomonas aeruginosa of an accepted model of bacterial permeability to beta-lactam antibiotics. Antimicrob Agents Chemother 35:916–921. doi:10.1128/AAC.35.5.916 1906695 PMC245129

[36] Simonet V, Malléa M, Pagès JM. 2000. Substitutions in the eyelet region disrupt cefepime diffusion through the Escherichia coli OmpF channel. Antimicrob Agents Chemother 44:311–315. doi:10.1128/AAC.44.2.311-315.2000 10639355 PMC89676

[37] Cai H, Rose K, Liang LH, Dunham S, Stover C. 2009. Development of a liquid chromatography/mass spectrometry-based drug accumulation assay in Pseudomonas aeruginosa. Anal Biochem 385:321–325. doi:10.1016/j.ab.2008.10.041 19032927

[38] Zhou Y, Joubran C, Miller-Vedam L, Isabella V, Nayar A, Tentarelli S, Miller A. 2015. Thinking outside the “bug”: a unique assay to measure intracellular drug penetration in gram-negative bacteria. Anal Chem 87:3579–3584. doi:10.1021/ac504880r 25753586

[39] Iyer R, Ye Z, Ferrari A, Duncan L, Tanudra MA, Tsao H, Wang T, Gao H, Brummel CL, Erwin AL. 2018. Evaluating LC-MS/MS to measure accumulation of compounds within bacteria. ACS Infect Dis 4:1336–1345. doi:10.1021/acsinfecdis.8b00083 29961312

[40] Modi N, Ganguly S, Bárcena-Uribarri I, Benz R, van den Berg B, Kleinekathöfer U. 2015. Structure, dynamics, and substrate specificity of the OprO porin from Pseudomonas aeruginosa. Biophys J 109:1429–1438. doi:10.1016/j.bpj.2015.07.035 26445443 PMC4601001

[41] Soundararajan G, Bhamidimarri SP, Winterhalter M. 2017. Understanding carbapenem translocation through OccD3 (OpdP) of Pseudomonas aeruginosa. ACS Chem Biol 12:1656–1664. doi:10.1021/acschembio.6b01150 28440622

[42] Dogan Guzel F, Pletzer D, Norouz Dizaji A, Al-Nahas K, Bajrai M, Winterhalter M. 2021. Towards understanding single-channel characteristics of OccK8 purified from Pseudomonas aeruginosa. Eur Biophys J 50:87–98. doi:10.1007/s00249-021-01498-5 33481046

[43] Golla VK, Prajapati JD, Joshi M, Kleinekathöfer U. 2020. Exploration of free energy surfaces across a membrane channel using metadynamics and umbrella sampling. J Chem Theory Comput 16:2751–2765. doi:10.1021/acs.jctc.9b00992 32167296

[44] Piselli C, Benz R. 2021. Fosmidomycin transport through the phosphate-specific porins OprO and OprP of Pseudomonas aeruginosa. Mol Microbiol 116:97–108. doi:10.1111/mmi.14693 33561903

[45] Lakaye B, Dubus A, Joris B, Frère JM. 2002. Method for estimation of low outer membrane permeability to beta-lactam antibiotics. Antimicrob Agents Chemother 46:2901–2907. doi:10.1128/AAC.46.9.2901-2907.2002 12183245 PMC127435

[46] National Committee for Clinical Laboratory Standards. 2020. Performance standards for antimicrobial susceptibility testing. In CLSI supplement M100, 30th ed. NCCLS, Wayne, PA, USA.

[47] Satake S, Yoneyama H, Nakae T. 1991. Role of OmpD2 and chromosomal beta-lactamase in carbapenem resistance in clinical isolates of Pseudomonas aeruginosa. J Antimicrob Chemother 28:199–207. doi:10.1093/jac/28.2.199 1778851

[48] Chandler CE, Horspool AM, Hill PJ, Wozniak DJ, Schertzer JW, Rasko DA, Ernst RK. 2019. Genomic and phenotypic diversity among ten laboratory isolates of Pseudomonas aeruginosa PAO1. J Bacteriol 201:e00595-18. doi:10.1128/JB.00595-18 30530517 PMC6379574

[49] Yu L, Cao Q, Chen W, Yang N, Yang CG, Ji Q, Wu M, Bae T, Lan L. 2022. A novel copper-sensing two-component system for inducing Dsb gene expression in bacteria. Sci Bull Sci Found Philipp 67:198–212. doi:10.1016/j.scib.2021.03.003 36546013

[50] Perron K, Caille O, Rossier C, Van Delden C, Dumas J-L, Köhler T. 2004. CzcR-CzcS, a two-component system involved in heavy metal and carbapenem resistance in Pseudomonas aeruginosa. J Biol Chem 279:8761–8768. doi:10.1074/jbc.M312080200 14679195

[51] Caille O, Rossier C, Perron K. 2007. A copper-activated two-component system interacts with zinc and imipenem resistance in Pseudomonas aeruginosa. J Bacteriol 189:4561–4568. doi:10.1128/JB.00095-07 17449606 PMC1913472

[52] Sobel ML, Hocquet D, Cao L, Plesiat P, Poole K. 2005. Mutations in PA3574 (nalD) lead to increased MexAB-OprM expression and multidrug resistance in laboratory and clinical isolates of Pseudomonas aeruginosa. Antimicrob Agents Chemother 49:1782–1786. doi:10.1128/AAC.49.5.1782-1786.2005 15855496 PMC1087681

[53] Ochs MM, McCusker MP, Bains M, Hancock RE. 1999. Negative regulation of the Pseudomonas aeruginosa outer membrane porin OprD selective for imipenem and basic amino acids. Antimicrob Agents Chemother 43:1085–1090. doi:10.1128/AAC.43.5.1085 10223918 PMC89115

[54] Acosta-Gutiérrez S, Bodrenko I, Ceccarelli M. 2021. The influence of permeability through bacterial porins in whole-cell compound accumulation. Antibiotics (Basel) 10:635. doi:10.3390/antibiotics10060635 34073313 PMC8226570

[55] Tommasi R, Iyer R, Miller AA. 2018. Antibacterial drug discovery: some assembly required. ACS Infect Dis 4:686–695. doi:10.1021/acsinfecdis.8b00027 29485271

[56] Glen KA, Lamont IL. 2021. β-lactam resistance in Pseudomonas aeruginosa: current status, future prospects. Pathogens 10:1638. doi:10.3390/pathogens10121638 34959593 PMC8706265

[57] Samanta S, Bodrenko I, Acosta-Gutiérrez S, D’Agostino T, Pathania M, Ghai I, Schleberger C, Bumann D, Wagner R, Winterhalter M, van den Berg B, Ceccarelli M. 2018. Getting drugs through small pores: exploiting the porins pathway in Pseudomonas aeruginosa. ACS Infect Dis 4:1519–1528. doi:10.1021/acsinfecdis.8b00149 30039960

[58] Montaner M, Lopez-Argüello S, Oliver A, Moya B. 2023. PBP target profiling by β-lactam and β-lactamase inhibitors in intact Pseudomonas aeruginosa: effects of the intrinsic and acquired resistance determinants on the periplasmic drug availability. Microbiol Spectr 11:e0303822. doi:10.1128/spectrum.03038-22 36475840 PMC9927461

[59] Jacoby GA. 2009. AmpC beta-lactamases. Clin Microbiol Rev 22:161–182, doi:10.1128/CMR.00036-08 19136439 PMC2620637

[60] Kojima S, Nikaido H. 2013. Permeation rates of penicillins indicate that Escherichia coli porins function principally as nonspecific channels. Proc Natl Acad Sci U S A 110:E2629–E2634. doi:10.1073/pnas.1310333110 23798411 PMC3710850

[61] Matsumura N, Minami S, Watanabe Y, Iyobe S, Mitsuhashi S. 1999. Role of permeability in the activities of beta-lactams against gram-negative bacteria which produce a group 3 beta-lactamase. Antimicrob Agents Chemother 43:2084–2086. doi:10.1128/AAC.43.8.2084 10428944 PMC89422

[62] Nikaido H. 1985. Role of permeability barriers in resistance to beta-lactam antibiotics. Pharmacol Ther 27:197–231. doi:10.1016/0163-7258(85)90069-5 2412244

[63] Gibson DG, Young L, Chuang R-Y, Venter JC, Hutchison CA III, Smith HO. 2009. Enzymatic assembly of DNA molecules up to several hundred kilobases. Nat Methods 6:343–345. doi:10.1038/nmeth.1318 19363495

[64] Bushnell B, Rood J, Singer E. 2017. BBMerge - accurate paired shotgun read merging via overlap. PLoS One 12:e0185056. doi:10.1371/journal.pone.0185056 29073143 PMC5657622

[65] Kearse M, Moir R, Wilson A, Stones-Havas S, Cheung M, Sturrock S, Buxton S, Cooper A, Markowitz S, Duran C, Thierer T, Ashton B, Meintjes P, Drummond A. 2012. Geneious Basic: an integrated and extendable desktop software platform for the organization and analysis of sequence data. Bioinformatics 28:1647–1649. doi:10.1093/bioinformatics/bts199 22543367 PMC3371832

[66] Ruijter JM, Ramakers C, Hoogaars WMH, Karlen Y, Bakker O, van den Hoff MJB, Moorman AFM. 2009. Amplification efficiency: linking baseline and bias in the analysis of quantitative PCR data. Nucleic Acids Res 37:e45. doi:10.1093/nar/gkp045 19237396 PMC2665230

[67] Hellemans J, Mortier G, De Paepe A, Speleman F, Vandesompele J. 2007. qBase relative quantification framework and software for management and automated analysis of real-time quantitative PCR data. Genome Biol 8:R19. doi:10.1186/gb-2007-8-2-r19 17291332 PMC1852402

[68] Vandesompele J, De Preter K, Pattyn F, Poppe B, Van Roy N, De Paepe A, Speleman F. 2002. Accurate normalization of real-time quantitative RT-PCR data by geometric averaging of multiple internal control genes. Genome Biol 3:RESEARCH0034. doi:10.1186/gb-2002-3-7-research0034 12184808 PMC126239

[69] Frère J-M, Nguyen-Distèche M, Coyette J, Joris B. 1992. Mode of action: interaction with the penicillin binding proteins, p 148–195. In Page M (ed), The chemistry of beta-lactams. Chapman and Hall, Glasgow, Scotland.

[70] Duval V, Swinnen M, Lepage S, Brans A, Granier B, Franssen C, Frère JM, Joris B. 2003. The kinetic properties of the carboxy terminal domain of the Bacillus licheniformis 749/I BlaR penicillin-receptor shed a new light on the derepression of beta-lactamase synthesis. Mol Microbiol 48:1553–1564. doi:10.1046/j.1365-2958.2003.03520.x 12791138

[71] Kolayli F, Karadenizli A, Savli H, Ergen K, Hatirnaz O, Balikci E, Budak F, Vahaboglu H. 2004. Effect of carbapenems on the transcriptional expression of the oprD, oprM and oprN genes in Pseudomonas aeruginosa. J Med Microbiol 53:915–920. doi:10.1099/jmm.0.45692-0 15314200

[72] Epp SF, Pechère J, Kok M. 2001. Raising antibodies against OprD, an outer membrane protein of Pseudomonas aeruginosa using translational fusions to MalE. J Microbiol Methods 46:1–8. doi:10.1016/s0167-7012(01)00236-6 11412908

