# Supplementary figures and images for: Outer membrane permeability of Pseudomonas aeruginosa through β-lactams: new evidence on the role of OprD and OpdP porins in antibiotic resistance
Source: Microbiol Spectr. 2025 Mar 4;13(4):e00495-24. doi: 10.1128/spectrum.00495-24 (PMC11960084; doi:10.1128/spectrum.00495-24)

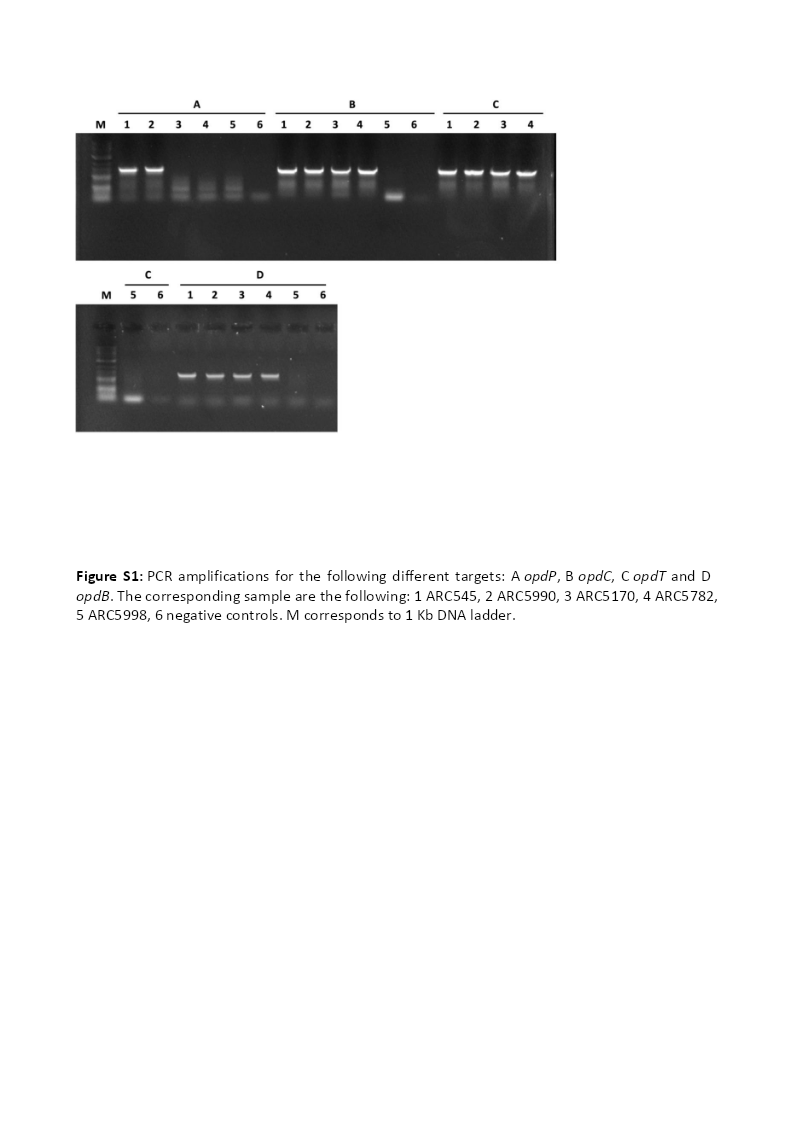

Supplement: Fig. S1 — Supplemental figure 1. [file spectrum.00495-24-s0001.tiff]
