# Supplementary material for: Outer membrane permeability of Pseudomonas aeruginosa through β-lactams: new evidence on the role of OprD and OpdP porins in antibiotic resistance
Source: Microbiol Spectr. 2025 Mar 4;13(4):e00495-24. doi: 10.1128/spectrum.00495-24 (PMC11960084; doi:10.1128/spectrum.00495-24)
Supplement: Supplemental material — Porin(s) deletion assessment; RT-PCR reference genes. [file spectrum.00495-24-s0004.docx]

**Supplemental information for: “Outer membrane permeability of *Pseudomonas aeruginosa*: elucidating the role of OprD and OpdP porins in antibiotic resistance”**

**Porin(s) deletion assessment**

A PCR-based screening was carried out on porin mutant strains to verify the presence/absence of the porins as previously described. Results are shown in Figure S1.

**RT-PCR reference genes**

The first goal to perform RT-PCRs was the determination of the most reliable reference genes for their stability at the different growth phases; we selected ten genes (*PA2875*, *PA3340*, *gyrA*, *recA*, *rho*, *proC*, *mreB* *cysG*, *gapA* and *mutL*), previously reported to be used as reference for qRT-PCR experiments and we analysed their relative expression in PAO1 in the early and late exponential phase and also during the stationary phase.

The quantification result is reported in figure S2.

We selected as references genes for the successive analysis *PA3340*, *gyrA* and *cysG* genes, on the basis of their stability during the bacterial growth and for their different relative expression. They have been in fact used as qRT-PCR internal controls.

**References (for tables S2 and S3)**

1. Shen J, Pan Y, Fang Y. 2015. Role of the Outer Membrane Protein OprD2 in Carbapenem-Resistance Mechanisms of *Pseudomonas aeruginosa*. PLoS One 10:e0139995.
2. Ocampo-Sosa AA, Cabot G, Rodríguez C, Roman E, Tubau F, Macia MD, Moya B, Zamorano L, Suárez C, Peña C, Domínguez MA, Moncalián G, Oliver A, Martínez-Martínez L; Spanish Network for Research in Infectious Diseases (REIPI). 2012. Alterations of OprD in carbapenem-intermediate and -susceptible strains of *Pseudomonas aeruginosa* isolated from patients with bacteremia in a Spanish multicenter study. Antimicrob Agents Chemother 56:1703-1713.
3. Caille O, Rossier C, Perron K. 2007. A copper-activated two-component system interacts with zinc and imipenem resistance in *Pseudomonas aeruginosa*. J Bacteriol 189:4561-4568.
4. Costaglioli P, Barthe C, Fayon M, Christoflour N, Bui S, Derlich L, Domblides P, Crouzet M, Vilain S, Garbay B. 2014. Selection of *Pseudomonas aeruginosa* reference genes for RT-qPCR analysis from sputum of cystic fibrosis patients. Mol Cell Probes 28:10-12.
5. Bragonzi A, Worlitzsch D, Pier GB, Timpert P, Ulrich M, Hentzer M, Andersen JB, Givskov M, Conese M, Doring G. 2005. Nonmucoid *Pseudomonas aeruginosa* expresses alginate in the lungs of patients with cystic fibrosis and in a mouse model. J Infect Dis 192:410-419.
6. Dumas JL, van Delden C, Perron K, Köhler T. 2006. Analysis of antibiotic resistance gene expression in *Pseudomonas aeruginosa* by quantitative real-time-PCR. FEMS Microbiol Lett 254:217-225.
7. Savli H, Karadenizli A, Kolayli F, Gundes S, Ozbek U, Vahaboglu H. 2003. Expression stability of six housekeeping genes: A proposal for resistance gene quantification studies of *Pseudomonas aeruginosa* by real-time quantitative RT-PCR. J Med Microbiol 52:403-408.
8. Gupta K, Marques CN, Petrova OE, Sauer K. 2013. Antimicrobial tolerance of *Pseudomonas aeruginosa* biofilms is activated during an early developmental stage and requires the two-component hybrid SagS. J Bacteriol 195:4975-4987.
9. Costaglioli P, Barthe C, Fayon M, Christoflour N, Bui S, Derlich L, Domblides P, Crouzet M, Vilain S, Garbay B. 2014. Selection of *Pseudomonas aeruginosa* reference genes for RT-qPCR analysis from sputum of cystic fibrosis patients. Mol Cell Probes 28:10-12.
