# Supplementary material for: Outer membrane permeability of Pseudomonas aeruginosa through β-lactams: new evidence on the role of OprD and OpdP porins in antibiotic resistance
Source: Microbiol Spectr. 2025 Mar 4;13(4):e00495-24. doi: 10.1128/spectrum.00495-24 (PMC11960084; doi:10.1128/spectrum.00495-24)
Supplement: Table S1 — Collection of plasmids used in this study. [file spectrum.00495-24-s0005.docx]

**Table S1:** Collection of plasmids used in this study.

| **Plasmid** | **Description** | **Resistance** | **Reference** |
| --- | --- | --- | --- |
| pKT240blaR | Shuttle vector for BlaR-CTD expression  in *E. coli* and *P. aeruginosa* | TET | (45) |
| pKT240neg | pKT240blaR depleted of *blaR-CTD* gene | TET | This study |
| pKT240blaR-gen | pKT240blaR added with *aac1* gene | TET, GEN | This study |
