# Supplementary material for: Outer membrane permeability of Pseudomonas aeruginosa through β-lactams: new evidence on the role of OprD and OpdP porins in antibiotic resistance
Source: Microbiol Spectr. 2025 Mar 4;13(4):e00495-24. doi: 10.1128/spectrum.00495-24 (PMC11960084; doi:10.1128/spectrum.00495-24)
Supplement: Table S2 — Collection of primers used in this study for PCR amplification and sequencing. [file spectrum.00495-24-s0006.docx]

**Table S2:** Collection of primers used in this study for PCR amplification and sequencing.

| **Primer** | **Sequence 5'=>3'** | **Reference** | **Use** |
| --- | --- | --- | --- |
| blaR-fw | CCATGCAAAAAGAAACACGC | This study | *blaR-CTD* amplification and sequencing |
| blaR-rev | TTCTTGCCTCCAAGTTCCGT | This study |  |
| blaR-seq-rev | ACAGGACAAAGCCCCCTGAGGCTG | This study | *blaR-CTD* sequencing |
| oprD-fw | ATGAAAGTGATGAAGTGGAGCG | 1 | *oprD* detection |
| oprD-rev | TTACAGGATCGACAGCGGATAG | 1 |  |
| oprD_flankF | CGGCTGAGGGGAAAGTCGCC | 2 | *oprD* sequencing |
| oprD_flankR | TACGCGGTCATTCTCGGGCG | 2 |  |
| opdB-fw | ATGATCCGCGTTCGACCGGTCG | This study | *opdB* detection |
| opdB-rev | TCAGAGCGAGCCTTTGAGTGGG | This study |  |
| opdC-fw | ATGAGGAATCTGTTCGCCTTGA | This study | *opdC* detection |
| opdC-rev | TCAGAACACGTCGATGGGATAG | This study |  |
| opdP-fw | GTGATGAGAAACCAACGTGTGA | This study | *opdP* detection |
| opdP-rev | TTACAGCAGGTTGAAGGGGAAG | This study |  |
| opdT-fw | ATGCAAGGGGATGGAAAGAAAC | This study | *opdT* detection |
| opdT-rev | TCAGAGGACTTGCAGCGGGTAT | This study |  |
| neg1-fw | AATAGGCGTATCACGAGGCCCTTTGCGTTCAGGCTGCTAAAGATG | This study | pKT240neg construction |
| neg1-rev | TCATGGTCTATTGCCTCCCG | This study |  |
| neg2-fw | CGGGAGGCAATAGACCATGA | This study | pKT240neg construction |
| neg2-rev | CAAAGGGCCTCGTGATACGC | This study |  |
| gmr_fw | GTTACGCCGTGGGTCGATGT | This study | pKT240blaR-gen construction |
| gmr_rev | AATTTACCGAACAACTCCGC | This study |  |
| gmr-inf-fw1 | GCGGAGTTGTTCGGTAAATTAGAAATTGCATCAACGCATA | This study | pKT240blaR-gen construction |
| gmr-inf-rev1 | GCGGGGTTTGGTGTGGGGTT | This study |  |
| gmr-inf-fw2 | AACCCCACACCAAACCCCGC | This study | pKT240blaR-gen construction |
| gmr-inf-rev2 | ACATCGACCCACGGCGTAACATGGAAGCCGGCGGCACCTC | This study |  |
|  |  |  |  |
