# Supplementary material for: Outer membrane permeability of Pseudomonas aeruginosa through β-lactams: new evidence on the role of OprD and OpdP porins in antibiotic resistance
Source: Microbiol Spectr. 2025 Mar 4;13(4):e00495-24. doi: 10.1128/spectrum.00495-24 (PMC11960084; doi:10.1128/spectrum.00495-24)
Supplement: Table S3 — Sequences and reaction efficiencies of quantitative RT-PCR primer pairs. [file spectrum.00495-24-s0007.docx]

**Table S3.** Sequences and reaction efficiencies of quantitative RT-PCR primer pairs.

| **Primer** | **Sequence 5'-3'** | **Reference** | **Reaction efficiency (RE)** | **Standard deviation (SD)** |
| --- | --- | --- | --- | --- |
| oprD-rt-fw | ATCTACCGCACAAACGATGAAGG | 3 | 1.925 | 0.0247 |
| oprD-rt-rev | GCCGAAGCCGATATAATCAAACG | 3 |  |  |
| opdB-rt-fw | GCTCAACCGCAACTACTTCC | This study | 1.753 | 0.0164 |
| opdB-rt-rev | GCGTCGGAATACTCGCTGGC | This study |  |  |
| opdC-rt-fw | GCTGAAGATCCGCGCCTTC | This study | 1.835 | 0.0178 |
| opdC-rt-rev | GCTGAGATGATGGCTGTCGC | This study |  |  |
| opdP-rt-fw | CAACACCGAATTCAAGGCC | This study | 1.923 | 0.0195 |
| opdP-rt-rev | CCGTACTCGGTGGTCATGTC | This study |  |  |
| opdT-rt-fw | GCTTCACCCTGGTCAACGAC | This study | 1.853 | 0.0181 |
| opdT-rt-rev | GAAGCTGGTGCCGGCGTAGT | This study |  |  |
| PA3340-rt-fw | GCTTGCAGTTCCTCAACGAG | 4 | 1.933 | 0.0229 |
| PA3340-rt-rev | CACCAGGAAATTCAGGTAGGG | 4 |  |  |
| gyrA-rt-fw | TGTGCTTTATGCCATGAGCGA | 5 | 1.934 | 0.0188 |
| gyrA-rt-rev | TCCACCGAACCGAAGTTGC | 5 |  |  |
| cysG-rt-fw | GCAGCAGCGCCGGGTGTTC | This study | 1.856 | 0.0223 |
| cysG-rt-rev | ACGTCGGCCTGCTGCATC | This study |  |  |
| rpsL-rt-fw | GCAAGCGCATGGTCGACAAGA | 6 | 1.883 | 0.0214 |
| rpsL-rt-rev | CGCTGTGCTCTTGCAGGTTGTGA | 6 |  |  |
| proC-rt-fw | CAGGCCGGGCAGTTGCTGTC | 7 | 1.919 | 0.0267 |
| proC-rt-rev | GGTCAGGCGCGAGGCTGTCT | 7 |  |  |
| gapA-rt-fw | CAACGACCAGAACCTCTCCG | This study | 1.884 | 0.0208 |
| gapA-rt-rev | ACCTGCACGGTGAGATCGAC | This study |  |  |
| mreB-rt-fw | CTGTCGATCGACCTGGG | 8 | 1.877 | 0.0184 |
| mreB-rt-rev | GATCACGCCGTCTTTCATCG | This study |  |  |
| rho-rt-fw | GCAACGGCTCCACCGAAGAC | This study | 1.901 | 0.0185 |
| rho-rt-rev | GTCACTTCCTCAGGGCGCTC | This study |  |  |
| recA-rt-fw | GAATCCTCGGGCAAGACCAC | This study | 1.910 | 0.0203 |
| recA-rt-rev | CACGTCGACCGCGTTGGAGC | This study |  |  |
| mutL-rt-fw | CAAGTTCCTGCGTGCCGAGA | This study | 1.651 | 0.0176 |
| mutL-rt-rev | CAGCGCCTGCTCGAGGAATG | This study |  |  |
| PA2875-rt-fw | AGTTTCCAGCGCATCCAGTT | 9 | 1.903 | 0.0205 |
| PA2875-rt-rev | CGGGATGGAAGACGAATTG | 9 |  |  |
| rpoS-rt-fw | CTCCCCGGGCAACTCCAAAAG | 7 | 1.898 | 0.0225 |
| rpoS-rt-rev | CGATCATCCGCTTCCGACCAG | 7 |  |  |
